# Effects of Se Application on Antioxidants and L‐DOPA Contents of Plant Parts in Faba Bean Cultivars (*Vicia faba* L.) in Salt Conditions

**DOI:** 10.1002/fsn3.72097

**Published:** 2026-07-12

**Authors:** Nurdoğan Topal, Ayşe Topuz

**Affiliations:** ^1^ Faculty of Agriculture, Department of Field Crops Uşak University Uşak Uşak Province Turkey

**Keywords:** antioxidant, fava bean, L‐DOPA, salt stress, selenium, total phenolics

## Abstract

This study aims to determine the effectiveness of exogenous selenium (Se) in regulating L‐DOPA and antioxidant profiles in different organs of *Vicia faba* L. at different salinity levels, along with identifying tissue‐specific metabolic changes. The research also aims to optimize selenium doses to increase the physiological resistance of the plant and its potential as a neuroprotective pharmaceutical substrate. For this purpose, the effects of Se application (doses: 0–0.05 to 0.1–0.15 mg/L) on L‐DOPA, Total Phenolic Content (TPC), Total Flavonoid Compound (TFC), and Antioxidant (DPPH%) contents in the leaves, flowers, and pods of two faba bean varieties grown under greenhouse conditions (at doses of 0–4‐8 dS/m) were investigated. Leaf, flower, and pod samples were taken during flowering and fruit formation periods, and L‐DOPA, TPC, TFC, and antioxidant content analyses were performed. Significant correlations (*p* < 0.01) were determined between salt and selenium doses in all components analyzed in the fruit, except for TPC. As a result of the analyses, the highest values were obtained for L‐DOPA at 0.15 mg/L Se application under 8 dS/m salt conditions, for antioxidant content at 0.15 mg/L Se application under 4 dS/m salt conditions, for TFC at 0.05 mg/L Se application under 8 dS/m salt conditions, and for TPC at 0.05 mg/L Se application under control (0 dS/m) salt conditions. To evaluate the molecular basis of the observed antioxidant potential, molecular docking was performed using AutoDock Vina to analyze the binding energy and polar/hydrophobic interactions of L‐DOPA to the active site of the pro‐oxidant enzyme NAD(P)H oxidase (PDB ID: 2CDU). The coupling analysis of L‐DOPA revealed a thermodynamically favorable binding energy (−6.7 kcal/mol) and the formation of stable hydrogen bonds with residues such as ASP282 and THR9; suggesting that L‐DOPA may function as a natural inhibitor of this pro‐oxidant enzyme. This makes L‐DOPA from *V. faba* a critical target for both plant physiology and human neuroprotective pharmacology. In summary, it is concluded that selenium application can help the plant cope with stress.

## Graphical abstract

Selenium‐Mediated Resilience in *Vicia faba* : The accumulation of secondary metabolites in *Vicia faba* L. represents a sophisticated and dynamic adaptive strategy in response to environmental perturbations. Our findings demonstrate that Selenium (Se) functions as a potent metabolic elicitor rather than a mere micronutrient, significantly modulating the biosynthesis of phenolic compounds and L‐DOPA. At optimized low dosages, specifically Se1 and Se2, selenium facilitates the upregulation of the phenylpropanoid biosynthetic pathway. This allows the plant to maintain a robust antioxidant defense system even under saline stress, as evidenced by the significant modulation of Total Phenolic Content (TPC) and Total Flavonoid Content (TFC) in foliar tissues. Such metabolic adjustments help the plant mitigate reactive oxygen species (ROS) production and maintain metabolic homeostasis instead of undergoing stress‐induced senescence. A critical observation in this study is the organ‐specific shift in metabolite allocation, particularly under severe salinity conditions (Salt 3). While the plant maintains higher antioxidant levels in vegetative tissues under mild stress, it undergoes a strategic “source‐to‐sink” translocation as stress intensifies, prioritizing the accumulation of L‐DOPA and phenolic antioxidants in the flowers. This prioritized biosynthesis in reproductive organs suggests a “reproductive safeguarding” mechanism intended to protect the plant's genetic progeny from ROS‐induced damage. Furthermore, the observed fluctuations in metabolite levels across treatments reflect a dose‐dependent, biphasic response to selenium; low concentrations act as bio‐stimulants, whereas higher doses (S4) under extreme salinity may interfere with primary metabolism or exacerbate oxidative stress. The empirical findings are scientifically validated at the molecular level through docking analysis, which provides a mechanistic framework for the observed antioxidant potency. The thermodynamically favorable binding energy of −6.7 kcal/mol between L‐DOPA and NAD(P)H oxidase indicates that L‐DOPA acts as a natural inhibitor of this pro‐oxidant enzyme. By stabilizing within the redox‐active region through hydrogen bonds and hydrophobic interactions, L‐DOPA effectively limits the initiation of ROS cascades. This specific interaction validates that the observed increases in total phenolics and flavonoids are synchronized with a targeted biochemical defense. While primary growth parameters are common indicators of salinity tolerance, this study prioritizes the metabolic plasticity of *Vicia faba* , as the synthesis of secondary metabolites often precedes visible morphological changes under stress. By utilizing HPLC‐UV to specifically quantify L‐DOPA—a specialized non‐protein amino acid and critical defense marker—we provide a nuanced understanding of how faba beans reprogram their secondary metabolism to maintain cellular integrity. Although specific antioxidant enzymes were not directly assayed, the integration of molecular docking with NAD(P)H oxidase serves as a biologically relevant alternative to explain the biochemical suppression of ROS generation, bridging the gap between total phenolic accumulation and specific enzymatic inhibition.

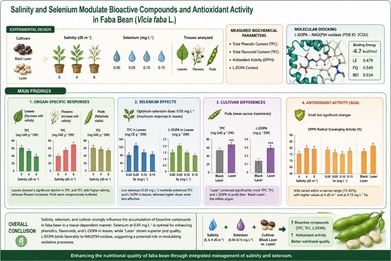

## 1 Introduction

Faba beans ( *Vicia faba* L.) are among the oldest crops in the world (Singh et al. 2013). According to FAOSTAT (2024), *Vicia faba* L. (faba bean, named also broad beans or horse bean, in Turkish Bakla) is the sixth among edible legumes in the world, and ranks fourth in Türkiye after chickpeas ( *Cicer arietinum* L.), lentils ( *Lens esculenta* Moench) and dry beans ( *Phaseolus vulgaris* L.). Faba beans are grown in many parts of the world due to their high nutritional value, medicinal effect, and effective biological nitrogen fixation and cultivated across various regions globally owing to their rich nutritional benefits, therapeutic properties, and proficient biological nitrogen fixation (Etemadi et al. 2019). This unique plant is among the few that contains L‐DOPA (L‐3.4‐dihydroxyphenylalanine) in its leaves, flowers, and fruit segments, which can aid in managing Parkinson's disease (PD), particularly prevalent in aging populations (Topal and Bozoğlu 2016). PD is a widespread neurodegenerative disorder marked by diminished muscle coordination, leading to limb tremors and instability of head posture (Hu et al. 2015). Diseases, damage, or physiological changes may occur in plants that will affect their development and survival during their lifetime (Shao et al. 2008). During their ontogenesis, plants face a dynamically changing environment defined by abiotic factors (e.g., light/dark, temperature, nutrient and water availability, and toxic compounds such as heavy metals) and biotic interactions (e.g., beneficial and pathogenic microbes, fungi, insects, other herbivores). Environmental perturbations which significantly disturb metabolism, development, and yield, are considered stress situations and cause stress responses in the biological system (Laxa et al. 2019). Among the abiotic stresses, the stress factor affecting the usable areas after the drought the most (26%) is mineral stress (Blum 1986). Salinity accounts for most of the mineral stress, and the world's salinity‐exposed area is more than 9 million hectares (Tuteja 2007).

Antioxidants, a notable class of compounds under investigation, have garnered scientific interest for their myriad benefits, including anti‐aging and anti‐inflammatory properties (Zehiroglu and Ozturk Sarikaya 2019). These substances are vital elements in the signaling and defense strategies of certain plants, where they serve as precursors to more complex compounds, regulators of plant growth, and protective measures against disease‐causing organisms and predators. The remarkable diversity of chemical structures and modifications found in various plant antioxidants renders them an invaluable source of intriguing compounds, capable of neutralizing reactive oxygen/nitrogen species (ROS/RNS) and activating intracellular signal cascades (Barreca 2020). Reactive oxygen species (ROS) emerge in plants during cellular metabolism. However, extreme environmental conditions such as drought, salinity, or flooding can lead to elevated ROS levels, resulting in the degradation of lipids, proteins, and nucleic acids, ultimately leading to plant demise. Increasing evidence indicates that ROS play a pivotal role as signaling molecules throughout the complete cell death pathway. Although ROS act as signaling agents, they can trigger an oxidative burst when there is a discrepancy between ROS production and their elimination. In addition to ROS, nitrogen species (RNS) significantly contribute to oxidative damage, tissue dysfunction, and serve as molecular signals. Therefore, cells establish a balanced system to mitigate the effects of ROS, incorporating an antioxidative defense system comprising enzymatic antioxidants like superoxide dismutase, catalase, and glutathione peroxidases, along with non‐enzymatic antioxidants, which work synergistically to lower oxidative stress (Kapoor et al. 2019). Polyphenols represent one of the most prevalent classifications of substances in plants. The term ‘polyphenol’ is reserved for compounds derived solely from the shikimate/phenylpropanoid and/or polyketide pathways, characterized by multiple phenolic units and devoid of nitrogen‐based functionalities (Hano and Tungmunnithum 2020). Phenolic compounds represent a crucial category of secondary metabolites in plants, fulfilling significant physiological functions throughout the plant's growth cycle. Phenolics are produced under both ideal and challenging conditions, playing key roles in developmental mechanisms such as cell division, hormonal regulation, photosynthesis, nutrient mineralization, and reproduction. In response to abiotic stresses, plants show heightened synthesis of polyphenols, including phenolic acids and flavonoids, which assist them in managing environmental adversities. The phenylpropanoid biosynthetic pathway becomes active during abiotic challenges (drought, heavy metal stress, salinity, extreme temperatures, and UV exposure), resulting in the buildup of diverse phenolic compounds that, among other roles, have the capacity to eliminate harmful reactive oxygen species (Sharma et al. 2019).

Recently, interest in selenium (Se) has surged as a crucial micronutrient, important not only for animals and humans but also for plants. Plants can absorb Selenium (Se) from the soil solution as selenate, selenite, and organic selenium through their roots. While the uptake of selenate and organic Se occurs actively, selenite is taken up through passive diffusion and can be hindered by phosphate (Sors et al. 2005). The absorption, transfer, and distribution of selenium fluctuate based on factors such as plant species, developmental phases, forms and concentrations of selenium, physiological conditions (like salinity and soil pH), and the presence of other compounds, transport protein activity, and the mechanisms of translocation within the plant (Gupta and Gupta 2017).

Application of Se under stress conditions results in alterations in the physiological and biochemical characteristics of plant cells concerning the effect of Se on the activation of both enzymatic and non‐enzymatic antioxidants under stress (Sieprawska et al. 2015).

Adapting to changing climate and environmental conditions and finding ways to increase productivity is always a moving target for us researchers. Applications that can help plants under stressful conditions will increase their importance over time. For this purpose, this study was carried out to determine the effects of selenium application on antioxidants and phenolic compounds such as L‐DOPA, Total Phenolic (TPC), and Total Flavonoid (TFC), which are important components of the health and plant defense system. The broad bean plant is one of the plants affected by salt stress. The study determined the effect of Se application on the examined compounds in fava bean plants grown under salt stress conditions. The objective of this study was to evaluate the efficacy of exogenous Selenium (Se) in modulating L‐DOPA and antioxidant profiles within different organs of *Vicia faba* L. under varying salinity levels, while investigating the molecular mechanism of L‐DOPA as a pro‐oxidant enzyme inhibitor. While salinity is known to impair morphological growth, the primary focus of this study was to evaluate the metabolic plasticity and functional quality of *Vicia faba* under stress. Unlike traditional growth‐oriented studies, we aimed to unravel how salt and selenium interact to modulate specific high‐value secondary metabolites, particularly L‐DOPA, which defines the plant's nutraceutical potential. We hypothesized that exogenous low‐dose Se could act as a metabolic elicitor to bypass salinity‐induced oxidative blocks, thereby systematically altering the source‐to‐sink partitioning of L‐DOPA.

## 2 Materials and Methods

### 2.1 Materials

Black Lazer and Lazer types obtained from Neobi local company were used in the study. In the selenium application, Sodium Selenate, anhydrous, 99.8 + % (metal basis) chemical with Alfa brand and CAS number 13410‐01‐0 was used. Table salt (NaCl) was used in salt applications. Pot work was carried out in polyethylene greenhouse conditions at Uşak University, Faculty of Agriculture (38°40′N, 29°24′E). The experiment was established and carried out in the form of factorial arrangements in random plots with 3 replications.

Samples were taken from the leaves in the vegetative period, from the flowers in the 50% flowering period, and from the fruits in the 50% fruit setting period, dried in an oven (at 70°C) and ground, and used to determine the L‐DOPA, Total Phenolic and flavonoid and antioxidant contents by measuring the required amounts.

### 2.2 Reagents

Glacial acetic acid (≥ 99%), acetonitrile (HPLC grade, ≥ 99%), and L‐DOPA standard were purchased from Sigma Chemical Co. Folin–Ciocalteu reagent, potassium dihydrogen phosphate, orthophosphoric acid, and triethylamine were purchased from Merck Company. The ultrapure water used in experimental studies with a conductivity of less than 0.05 μS cm^−1^ was produced using a Milli‐Q System. All other chemicals were of analytical purity.

### 2.3 Equipment

Shimadzu 1800 model UV–Vis Spectrophotometer, Agilent 1260 model high‐performance liquid chromatograph instrument, Mettler Toledo Precision Balance, and Wisd Ultrasonic Bath.

### 2.4 Methods

In the study, two bean varieties (Black Lazer, Lazer), three different doses of salt (0–4‐8 dS/m), and four different doses of selenium (Se) (0–0.05 to 0.1–0.15 mg/L) were used. In the study, 5 seeds were planted in each pot (3 L). Before planting, basic (NPK) fertilizer was applied to each pot. The salinity doses (0, 4, and 8 dS/m) were selected to simulate a gradient of environmental stress, ranging from control conditions to severe salinity, considering that *Vicia faba* L. is classified as moderately salt sensitive. The concentration of 8 dS/m was specifically chosen to push the plant's physiological limits and observe the protective role of Selenium under peak oxidative stress. For Selenium application, the dosages (0, 0.05, 0.1, and 0.15 mg/L) were determined based on previous studies demonstrating that trace levels of Selenium effectively upregulate the antioxidant defense system and L‐DOPA biosynthesis while avoiding the biphasic decline and phytotoxicity often associated with higher concentrations. For 100 ppm of nitrogen fertilizer application, 317 g of Ca (NO_3_)_2_ was weighed and dissolved in 1250 mL of distilled water. 5 mL of this solution was applied to each pot. Phosphorus application for 100 ppm, 164.63 g of KH_2_PO_4_ fertilizer was weighed, dissolved in 1250 mL of distilled water and 5 mL was applied to each pot. 125 ppm potassium was applied at the same time as the KH_2_PO_4_ application. One day after planting, the first salt application was applied to each pot as 300 mL. The second salt application was applied approximately one month after planting and 200 mL per pot. 45 days after planting, solutions containing different amounts of Se were applied to the plants by spraying method once a week for four weeks.

### 2.5 Extraction

All extraction methods were performed according to Topal et al. (2020). Dried into extraction vessels. To each sample, 50 mL of a 70% methanol solution in ultra‐pure water was added, resulting in a final sample concentration of 10 mg/mL (w/v). The mixture was then subjected to extraction in an ultrasonic bath for 15 min to facilitate the release of secondary metabolites. Following the extraction period, the crude extract was filtered through white‐band (Whatman No: 42) filter paper to remove particulate matter. To preserve the integrity of heat‐sensitive bioactive compounds and prevent the degradation of L‐DOPA, the resulting liquid filtrates were used directly for subsequent analyses without further concentration or solvent evaporation. Specific aliquots were taken from this 10 mg/mL stock for the determination of TPC (500 μL), TFC (50 μL), and antioxidant activity (300 μL), while the remaining filtrate was used for HPLC‐UV analysis.

### 2.6 Total Phenolic Content (TPC)

The samples' total phenolic content (TPC) was determined using the Folin‐Ciocalteau method (Petrović et al. 2022). After adding 500 μL of sample extract, 250 μL of folin–Ciocalteu reagent, 7250 μL of ultrapure water to a 10 mL test tube, it was kept in the dark for 5 min. 2000 μL of 7.5% Na_2_CO_3_ solution was added and kept in a dark environment for 30 min. After 30 min of incubation, the absorbance value against ultrapure water was measured at 765 nm with a spectrophotometer (Shimadzu UV‐1800 spectrophotometer, Japan) instrument. TPC was expressed as mg gallic acid equivalent (GAE) per 1.00 g dried plant material.

### 2.7 Total Flavonoid Content (TFC)

The samples' total flavonoid content (TFC) was determined using the aluminum chloride colorimetric method (Lopes et al. 2022). For this, 50 μL of sample extract, 950 μL of methanol, 6400 μL of ultra‐pure water, 300 μL of NaNO2 solution (5% in ultra‐pure water), and 300 μL of AlCl3 solution (10% in ultra‐pure water) were added to a 10 mL test tube and kept in a dark environment for 5 min. Then, 2000 μL of NaOH solution (4% in ultra‐pure water) was added and it was kept in the dark for 15 min again. The absorbance of the mixture against ultrapure water at a wavelength of 510 nm was measured using a spectrophotometer (Shimadzu UV‐1800 spectrophotometer, Japan). Total flavonoid content was expressed as mg quercetin equivalent (QE) per 1 g dried plant material.

### 2.8 Antioxidant Activity (DPPH Radical Scavenging Activity)

Antioxidant activity was determined using the DPPH (2,2‐diphenyl‐1‐picrylhydrazil) radical method (Ebrahimzadeh et al. 2008). For this, 0.0024 g of DPPH was precisely weighed, and a stock solution of DPPH reagent (6 × 10–5 M) was prepared by dissolving in 100 mL of methanol. A working solution of DPPH with a concentration (40 mg L^−1^) was prepared from the stock reagent solution by diluting it with methanol. 300 μL of sample extract and 5700 μL of DPPH working solution were mixed in a 10 mL test tube. The mixture was incubated for 60 min at room temperature in a dark environment. The absorbance of the reaction mixture against ultrapure water was measured at 517 nm using a spectrophotometer (Shimadzu UV‐1800 spectrophotometer, Japan). On the other hand, a control solution without sample extract was prepared and its absorbance against ultrapure water was measured at 517 nm in a spectrophotometer device. The antioxidant activity was calculated as:

$$\begin{array}{c} \text{DPPH Radical Scavenging Activity}\;\left(\%\right)\\ =\left(\mathrm{AC}\left(O\right)517-\mathrm{AA}\left(t\right)517\right)/\mathrm{AC}\left(O\right)517\times 100 \end{array}$$

where AC(O)517 is the absorbance of the control at *t* = 0 min and AA(*t*)517 is the absorbance of the antioxidant at *t* = 1 h.

### 2.9 Analytical Validation and Calibration

The analytical methods were validated by determining the Limit of Detection (LOD), Limit of Quantitation (LOQ), and linearity. Calibration curves were constructed using five concentration levels for each standard. The comprehensive performance of the calibration and validation metrics across the evaluated phytochemical assays is systematically documented in Table 1. The LOD and LOQ were calculated based on the standard deviation of the response (σ) and the slope (S) of the calibration curve using the formulas outlined by Rao (2018):

$$\mathrm{LOD}=3.3\times \frac{\sigma_{\text{blank}}}{{\text{slope}}_{m}}$$

**TABLE 1:** Calibration and validation parameters for phytochemical analyses.

Component	Standard	Regression equation	*R* ^2^	LOD (mg/L)	LOQ (mg/L)
TPC	Gallic Acid	*y* = 0.0012*x* + 0.045	0.9982	0.45	0.36
TFC	Quercetin	*y* = 0.0021*x* + 0.012	0.9991	0.97	0.32
L‐DOPA	L‐DOPA	*y* = 15.42*x*−3.21	0.9998	0.36	0.12

To guarantee the analytical reliability of L‐DOPA quantification across highly divergent plant organs, the validation parameters (LOD and LOQ) were strictly optimized. This optimization was particularly crucial for the pod matrix, where baseline L‐DOPA concentrations are inherently lower compared to follicular and floral tissues (Tesoro et al. 2022). The high chromatographic selectivity of the optimized C18 stationary phase, coupled with a low pH mobile phase (pH 2.30), effectively resolved L‐DOPA from confounding matrix interferences, ensuring stable peak integration and reproducible recovery rates even at near‐limit quantitation boundaries (Bulduk and Topal 2021).

### 2.10 L‐DOPA Content

The L‐DOPA contents of the samples were determined using an Agilent 1260 model HPLC (Agilent Technologies, USA) instrument equipped with a UV detector and Chemstation software. Chromatographic analyzes were performed on an Ace C18 (250 × 4.6 mm, 5 μm) column. Potassium dihydrogen phosphate solution with a concentration of 50 mM, whose pH was adjusted to 2.30 with phosphoric acid, was used as the mobile phase. The mobile phase flow rate was 1.2 mL min^−1^, the column temperature was 30°C, and the detector wavelength was 280 nm (Bulduk and Topal 2021).

### 2.11 L‐DOPA Molecular Docking

The selection of PDB ID: 2CDU as the target structure is based on the direct role of NADPH oxidase 4 (NOX4) in the intracellular generation of reactive oxygen species (ROS). NOX4 is recognized as a key enzymatic source of oxidative stress, making it a biologically relevant target for evaluating antioxidant mechanisms. Since antioxidant activity is fundamentally associated with the suppression or reduction of free radical formation, ligands capable of binding to the active site of NOX4 are expected to potentially limit ROS production. Therefore, the use of the 2CDU structure in molecular docking analyses provides a mechanistically meaningful framework for explaining the antioxidant potential of the investigated compounds at the molecular level.

Molecular docking analyses were performed to evaluate the antioxidant potential of the investigated compounds at the molecular level. To model antioxidant‐related interactions, NAD(P)H oxidase was selected as the target protein due to its direct involvement in the generation of intracellular reactive oxygen species (ROS). In this context, the crystal structure of the water‐forming NAD(P)H oxidase from *Lactobacillus sanfranciscensis* (PDB ID: 2CDU) was employed. This structure provides a biologically relevant redox‐active binding environment, making it suitable for elucidating ligand–protein interactions associated with antioxidant mechanisms.

The selected crystal structure contains levodopa as the co‐crystallized ligand, and the docking grid was defined based on the native ligand binding site. Accordingly, the grid box was centered at *x* = 11, *y* = 7, and *z* = 50 to ensure accurate sampling of the active region. The protein structure was retrieved from the RCSB Protein Data Bank and prepared for docking using AutoDock Tools (version 4.2). Protein preparation steps included removal of crystallographic water molecules, addition of polar hydrogen atoms, assignment of Gasteiger partial charges, and conversion of the structure into PDBQT format.

Ligand structures were prepared using PyRx in accordance with the AutoDock Vina workflow, including geometry optimization while preserving torsional flexibility. Docking simulations were carried out using AutoDock Vina, generating multiple binding conformations for each ligand. The binding pose with the lowest predicted binding free energy was selected for subsequent analysis (Trott and Olson 2010). Detailed protein–ligand interactions, including hydrogen bonds and hydrophobic contacts, were analyzed using Discovery Studio Visualizer (Aytar 2024).

### 2.12 Statistical Analyses

All biochemical analyses were performed on three independent biological replicates (*n* = 3) per treatment combination, with each replicate measured in technical triplicate; means of technical replicates were used as the observational unit, giving a final design of 72 experimental observations per response variable (2 cultivars × 3 salinity levels × 4 Se doses × 3 biological replicates). Statistical analyses were conducted in IBM SPSS Statistics v.27.0 (IBM Corp., Armonk, NY, USA) and R v.4.3.2 (R Core Team 2023).

Prior to parametric analysis, the normality of residuals was assessed by the Shapiro–Wilk test, and the homogeneity of variances was assessed by Levene's test (*α* = 0.05). When assumptions were met, data were analyzed by three‐way analysis of variance (ANOVA) with cultivar (V), salinity (Salt), and selenium dose (Se) as fixed factors, separately for each plant organ (leaves, flowers, pods), thereby allowing the organ‐specific evaluation requested during peer review. The full statistical model was:

$$\begin{array}{c} Y_{\text{ijkl}}=\mu +V_{i}+\mathrm{Sal}t_{j}+Se_{k}+{\left(V\times \text{Salt}\right)}_{\mathrm{ij}}+{\left(V\times \mathrm{Se}\right)}_{\mathrm{ik}}+{\left(\text{Salt}\times \mathrm{Se}\right)}_{\mathrm{jk}}\\ \;+{\left(V\times \text{Salt}\times \mathrm{Se}\right)}_{\mathrm{ijk}}+\varepsilon_{\text{ijkl}} \end{array}$$

where *Y*_ijkl_ is the observed value of the response variable, *μ* is the overall (grand) mean, *V*_*i*_ is the fixed effect of the *i*‐th cultivar (*i* = 1, 2), Salt_*j*_ is the fixed effect of the *j*‐th salinity level (*j* = 1, 2, 3), Se_*k*_ is the fixed effect of the *k*‐th selenium dose (*k* = 1, 2, 3, 4), (*V* × Salt)_*ij*_, (*V* × Se)_*ik*_, and (Salt × Se)_*jk*_ are the two‐way interaction terms, (V × Salt × Se)_*ijk*_ is the three‐way interaction term, and *ε*_*ijkl*_ is the residual error of the *l*‐th replicate of the *ijk*‐th treatment combination (*l* = 1, 2, 3), assumed to be independently and identically distributed (*ε* ~ *N*(0, *σ* ^2^)). Type III sums of squares were used to accommodate balanced factorial design conventions and to avoid order‐of‐entry effects. When ANOVA detected significant effects (*p* < 0.05), pairwise mean comparisons were performed using Tukey's honestly significant difference (HSD) post hoc test (*α* = 0.05).

### 2.13 Multivariate Analyses

Two complementary multivariate approaches were applied to the standardized (z‐score) 12‐variable dataset to characterize the integrated biochemical phenotype across treatments: (i) Principal Component Analysis (PCA) was performed using the FactoMineR (Lê et al. 2008) and factoextra (Kassambara and Mundt 2016) packages in R to identify the dominant axes of biochemical variation. Components were retained based on the Kaiser criterion (eigenvalue > 1) supplemented by inspection of the scree plot. Variable loadings, contributions (%) to each axis, and quality of representation (cos^2^) were extracted; a biplot was constructed to simultaneously project sample scores and variable loadings, with 90% confidence ellipses drawn around salinity‐level groups. (ii) Hierarchical Cluster Analysis (HCA) was performed on the 24 treatment‐mean profiles using Ward's minimum‐variance linkage (ward. D2 algorithm; Murtagh and Legendre 2014) on Euclidean distances of the standardized data. The optimal number of clusters was determined by visual inspection of the dendrogram, the elbow of the within‐cluster sum‐of‐squares plot, and biological interpretability; four clusters were retained. Results were visualized as a row‐ and column‐clustered heatmap using the pheatmap package (Kolde 2019), with row annotations indicating cultivar, salinity level, selenium dose, and cluster membership. A significance threshold of *α* = 0.05 was applied throughout. Data are presented as means ± standard deviation (SD) unless otherwise stated.

## 3 Results

### 3.1 Three‐Way ANOVA of the Four Biochemical Parameters

The factorial analysis confirmed that salinity, selenium dose, and cultivar were significant sources of variation for almost every parameter–organ combination, with the pattern of effects differing markedly between tissues (Table 2). Salinity and selenium acted as the dominant factors in leaves and flowers, while cultivar mattered most in pods. The interaction terms were significant in 31 of 48 cases, which justified looking at the three‐way interaction means rather than relying on main effects alone (Tables 3b–d, 4b–d, 5b–d, 6b–d).

**TABLE 2:** Three‐way analysis of variance (*F* values and significance levels) for the effects of cultivar (V), salinity (Salt), selenium dose (Se), and their interactions on total phenolic content (TPC), total flavonoid content (TFC), antioxidant activity (AOA), and L‐DOPA content in leaves, flowers, and pods of two faba bean ( *Vicia faba* L.) cultivars.

Source of variation	df	Total Phenolic Content (TPC)			Total Flavonoid Content (TFC)		
		mg GAE g^−1^ DW			mg CE g^−1^ DW		
		Leaves	Flowers	Pods	Leaves	Flowers	Pods
Cultivar (V)	1	2.86^ns^	73.51***	16.63***	50.36***	60.06***	24.88***
Salinity (Salt)	2	23.88***	44.76***	2.36^ns^	177.14***	271.12***	5.51**
Selenium (Se)	3	11.88***	65.67***	0.84^ns^	192.64***	97.32***	14.79***
*V* × Salt	2	1.76^ns^	4.18*	12.77***	6.36**	22.93***	6.38**
*V* × Se	3	0.83^ns^	23.40***	2.36^ns^	8.87***	26.40***	17.08***
Salt × Se	6	5.36***	6.53***	9.65***	58.48***	42.57***	13.24***
*V* × Salt × Se	6	1.65^ns^	13.29***	6.35***	8.03***	71.87***	4.42**
Error (residual)	48	MSE = 70.42	MSE = 42.64	MSE = 28.68	MSE = 50.60	MSE = 61.34	MSE = 52.88

		Antioxidant Activity (AOA)			L‐DOPA content		
		% DPPH scavenging			mg g^−1^ DW		
		Leaves	Flowers	Pods	Leaves	Flowers	Pods
Cultivar (V)	1	2784.40***	751.54***	9114.69***	0.04^ns^	2.46^ns^	17.63***
Salinity (Salt)	2	1193.87***	2473.10***	4545.83***	19.63***	45.17***	14.41***
Selenium (Se)	3	1615.49***	5701.12***	5421.98***	7.15***	17.83***	9.23***
*V* × Salt	2	1026.44***	2704.96***	4435.82***	1.20^ns^	8.70***	0.13^ns^
*V* × Se	3	317.27***	4020.66***	4576.09***	3.13*	6.58***	4.49**
Salt × Se	6	2731.47***	6006.06***	3341.72***	0.76^ns^	2.73*	3.08*
*V* × Salt × Se	6	2401.11***	2677.32***	4497.67***	2.85*	0.94^ns^	3.52**
Error (residual)	48	MSE = 0.02	MSE = 0.01	MSE = 0.05	MSE = 0.22	MSE = 0.96	MSE = 0.01

*Note:* *F* values were computed from a three‐way factorial ANOVA model (Type III sums of squares) with cultivar (V; 2 levels), salinity (Salt; 3 levels: 0, 4, 8 dS m^−1^), and selenium dose (Se; 4 levels: 0, 0.05, 0.10, 0.15 mg L^−1^) as fixed factors. *n* = 9 measurements per cell (3 biological × 3 technical replicates); total *N* = 72 per response variable. Degrees of freedom (df) are common across all response variables given the balanced factorial design. Significance levels: ***p < 0.001; **p < 0.01; *p < 0.05; ns, not significant (*p* ≥ 0.05). MSE, mean square error (residual variance). The exceptionally high *F*‐statistics observed for DPPH antioxidant activity (AOA) reflect the very low within‐cell variance (MSE < 0.06) of these particular measurements, which arises from the high technical reproducibility of the spectrophotometric DPPH assay performed in immediate succession on the same instrument; this is to be interpreted alongside the effect‐size and biological‐magnitude considerations presented in Tables 3, 4, 5, 6.

Abbreviations: CE, catechin equivalents; DW, dry weight; GAE, gallic acid equivalents.

**TABLE 3:** Total phenolic content (TPC, mg GAE g^−1^ DW).

(a) Main effects of salinity, selenium dose, and cultivar				
Factor	Level	Leaves	Flowers	Pods
Salinity (dS m^−1^)	0	66.96 ± 10.06^a^	45.55 ± 15.96^b^	32.27 ± 8.21^a^
	4	53.88 ± 15.00^b^	46.38 ± 23.51^b^	29.16 ± 7.83^a^
	8	51.37 ± 9.27^b^	61.40 ± 12.23^a^	31.81 ± 10.74^a^
Se dose (mg L^−1^)	0.00	52.64 ± 11.21^b^	51.93 ± 13.27^a^	30.50 ± 8.33^a^
	0.05	67.37 ± 13.80^a^	52.60 ± 18.26^a^	29.80 ± 7.44^a^
	0.10	53.41 ± 13.48^b^	34.77 ± 12.88^b^	31.61 ± 10.72^a^
	0.15	56.18 ± 10.48^b^	65.14 ± 18.69^a^	32.41 ± 9.71^a^
Cultivar	Black Lazer	59.07 ± 13.56^a^	57.71 ± 19.58^a^	28.50 ± 8.85^b^
	Lazer	55.73 ± 13.31^a^	44.51 ± 16.28^b^	33.65 ± 8.50^a^

(b) Three‐way interaction means (Cultivar × Salinity × Se dose) for leaves (means ± standard deviation (*n* = 3))					
Cultivar	Salinity (dS m^−1^)	Se dose (mg L^−1^)			
		0.00	0.05	0.10	0.15
Black Lazer	0	64.4 ± 11.0	80.9 ± 10.0	70.0 ± 4.5	68.1 ± 8.8
	4	45.3 ± 10.0	74.4 ± 12.4	45.4 ± 6.2	57.7 ± 5.2
	8	52.3 ± 4.1	45.3 ± 10.0	51.0 ± 0.0	54.3 ± 2.0
Lazer	0	64.3 ± 4.9	69.6 ± 2.0	66.7 ± 10.9	51.8 ± 3.9
	4	47.9 ± 10.0	74.1 ± 5.0	36.4 ± 4.7	49.8 ± 10.0
	8	41.8 ± 2.1	59.9 ± 0.1	51.0 ± 11.0	55.5 ± 20.0

(c) Three‐way interaction means (Cultivar × Salinity × Se dose) for flowers (means ± standard deviation (*n* = 3))					
Cultivar	Salinity (dS m^−1^)	Se dose (mg L^−1^)			
		0.00	0.05	0.10	0.15
Black Lazer	0	59.3 ± 4.0	61.7 ± 4.2	32.8 ± 7.3	65.6 ± 4.8
	4	27.4 ± 5.4	61.9 ± 6.0	28.5 ± 4.7	94.0 ± 2.1
	8	59.9 ± 1.0	70.1 ± 10.0	56.6 ± 1.0	74.5 ± 5.4
Lazer	0	44.0 ± 3.1	38.7 ± 9.1	18.5 ± 2.9	43.7 ± 4.0
	4	57.4 ± 3.5	22.4 ± 1.0	32.3 ± 9.0	47.1 ± 6.0
	8	63.6 ± 3.0	60.8 ± 11.0	39.8 ± 4.0	65.8 ± 17.2

(d) Three‐way interaction means (Cultivar × Salinity × Se dose) for pods (means ± standard deviation (*n* = 3))					
Cultivar	Salinity (dS m^−1^)	Se dose (mg L^−1^)			
		0.00	0.05	0.10	0.15
Black Lazer	0	36.5 ± 7.0	31.7 ± 0.9	23.5 ± 4.4	23.5 ± 4.7
	4	20.7 ± 2.3	39.7 ± 5.7	37.4 ± 5.7	25.7 ± 6.1
	8	27.7 ± 6.0	17.3 ± 1.9	19.1 ± 0.0	39.4 ± 6.0
Lazer	0	39.7 ± 10.9	31.6 ± 1.1	41.4 ± 1.1	30.3 ± 9.6
	4	27.9 ± 5.9	28.0 ± 3.1	23.4 ± 2.0	30.6 ± 10.0
	8	30.6 ± 0.2	30.6 ± 5.0	44.9 ± 5.0	45.0 ± 1.0

*Note:* Values are means ± standard deviation (*n* = 3 biological replicates, each measured in triplicate; total *n* = 9 per cell). Within each tissue column, means within the same factor block (Salinity, Selenium, or Cultivar) followed by different lowercase letters are significantly different at *p* < 0.05 according to Tukey's HSD post hoc test. The full three‐way ANOVA results (main and interaction effects with *F* and *p* values) are reported in Table 2.

Abbreviations: DW, dry weight; GAE, gallic acid equivalents.

**TABLE 4:** Total flavonoid content (TFC, mg CE g^−1^ DW).

(a) Main effects of salinity, selenium dose, and cultivar				
Factor	Level	Leaves	Flowers	Pods
Salinity (dS m^−1^)	0	88.84 ± 25.63^a^	63.58 ± 36.17^b^	62.10 ± 17.63^a^
	4	72.00 ± 40.31^a^	75.89 ± 34.00^b^	64.18 ± 15.33^a^
	8	50.29 ± 13.06^b^	114.07 ± 27.71^a^	57.38 ± 10.71^a^
Se dose (mg L^−1^)	0.00	62.69 ± 21.35^b^	94.57 ± 37.82^a^	69.86 ± 11.89^a^
	0.05	103.89 ± 34.09^a^	93.76 ± 38.03^a^	54.46 ± 14.33^b^
	0.10	64.86 ± 28.09^b^	57.23 ± 29.37^b^	62.32 ± 16.69^a^
	0.15	50.08 ± 15.63^b^	92.49 ± 39.19^a^	58.24 ± 12.82^a^
Cultivar	Black Lazer	76.33 ± 27.99^a^	91.67 ± 41.98^a^	56.94 ± 16.11^b^
	Lazer	64.43 ± 35.60^a^	77.36 ± 34.73^a^	65.49 ± 12.38^a^

(b) Three‐way interaction means (Cultivar × Salinity × Se dose) for leaves (means ± standard deviation (*n* = 3))					
Cultivar	Salinity (dS m^−1^)	Se dose (mg L^−1^)			
		0.00	0.05	0.10	0.15
Black Lazer	0	68.1 ± 8.0	120.6 ± 0.1	104.1 ± 0.1	69.4 ± 2.5
	4	57.3 ± 2.3	137.1 ± 0.1	57.6 ± 7.0	69.6 ± 0.1
	8	60.6 ± 0.1	69.6 ± 9.0	57.6 ± 3.0	44.1 ± 5.0
Lazer	0	101.9 ± 19.0	107.2 ± 13.0	95.1 ± 6.0	44.1 ± 4.0
	4	44.0 ± 1.1	137.2 ± 4.0	29.1 ± 10.0	44.1 ± 7.0
	8	44.1 ± 0.1	51.6 ± 2.0	45.7 ± 13.9	29.1 ± 5.1

(c) Three‐way interaction means (Cultivar × Salinity × Se dose) for flowers (means ± standard deviation (*n* = 3))					
Cultivar	Salinity (dS m^−1^)	Se dose (mg L^−1^)			
		0.00	0.05	0.10	0.15
Black Lazer	0	117.9 ± 3.9	86.8 ± 3.9	44.8 ± 4.3	23.1 ± 0.1
	4	35.0 ± 7.1	60.6 ± 8.0	84.6 ± 1.0	121.4 ± 10.0
	8	147.6 ± 3.0	140.3 ± 7.2	101.1 ± 0.8	126.7 ± 11.0
Lazer	0	80.1 ± 3.0	36.6 ± 13.0	23.1 ± 6.0	86.1 ± 2.0
	4	77.1 ± 2.0	125.1 ± 0.1	29.0 ± 1.9	74.1 ± 7.0
	8	99.6 ± 1.9	113.1 ± 9.0	60.6 ± 9.0	123.6 ± 13.0

(d) Three‐way interaction means (Cultivar × Salinity × Se dose) for pods (means ± standard deviation (*n* = 3))					
Cultivar	Salinity (dS m^−1^)	Se dose (mg L^−1^)			
		0.00	0.05	0.10	0.15
Black Lazer	0	87.4 ± 4.9	38.7 ± 3.8	37.3 ± 3.6	54.4 ± 12.5
	4	71.0 ± 4.1	50.1 ± 8.9	77.2 ± 0.5	57.6 ± 8.0
	8	63.5 ± 1.0	39.6 ± 0.3	47.1 ± 0.1	58.6 ± 7.5
Lazer	0	72.6 ± 6.0	66.3 ± 10.8	68.1 ± 6.0	71.1 ± 6.0
	4	71.1 ± 11.0	71.2 ± 5.0	77.1 ± 17.0	38.1 ± 1.0
	8	53.3 ± 7.2	60.6 ± 9.0	66.7 ± 6.0	69.6 ± 3.0

*Note:* Values are means ± standard deviation (*n* = 3 biological replicates, each measured in triplicate; total *n* = 9 per cell). Within each tissue column, means within the same factor block (Salinity, Selenium, or Cultivar) followed by different lowercase letters are significantly different at *p* < 0.05 according to Tukey's HSD post hoc test. The full three‐way ANOVA results (main and interaction effects with *F* and *p* values) are reported in Table 2.

Abbreviations: CE, Catechin Equivalent; DW, dry weight.

**TABLE 5:** Antioxidant activity (% DPPH radical scavenging).

(a) Main effects of salinity, selenium dose, and cultivar				
Factor	Level	Leaves	Flowers	Pods
Salinity (dS m^−1^)	0	77.87 ± 1.97^a^	78.93 ± 3.99^a^	75.30 ± 11.21^b^
	4	79.86 ± 5.27^a^	80.68 ± 5.30^a^	81.24 ± 2.83^a^
	8	78.59 ± 2.21^a^	80.98 ± 1.11^a^	77.22 ± 8.16^a^
Se dose (mg L^−1^)	0.00	76.97 ± 1.96^b^	81.49 ± 1.18^a^	80.25 ± 1.99^a^
	0.05	78.84 ± 1.35^a^	78.77 ± 4.72^b^	76.18 ± 7.88^a^
	0.10	80.23 ± 6.21^a^	78.31 ± 4.37^b^	73.43 ± 13.39^b^
	0.15	79.04 ± 1.54^a^	82.21 ± 3.15^a^	81.81 ± 2.24^a^
Cultivar	Black Lazer	79.66 ± 4.51^a^	80.55 ± 4.36^a^	75.44 ± 11.24^b^
	Lazer	77.89 ± 1.86^b^	79.84 ± 3.49^a^	80.40 ± 2.29^a^

(b) Three‐way interaction means (Cultivar × Salinity × Se dose) for leaves (means ± standard deviation (*n* = 3))					
Cultivar	Salinity (dS m^−1^)	Se dose (mg L^−1^)			
		0.00	0.05	0.10	0.15
Black Lazer	0	78.6 ± 0.4	78.6 ± 0.1	78.4 ± 0.0	79.6 ± 0.1
	4	76.1 ± 0.0	79.4 ± 0.2	93.2 ± 0.2	77.7 ± 0.2
	8	78.7 ± 0.2	79.9 ± 0.0	74.2 ± 0.0	81.1 ± 0.0
Lazer	0	73.4 ± 0.1	76.2 ± 0.0	79.0 ± 0.0	78.8 ± 0.0
	4	78.4 ± 0.0	78.5 ± 0.1	78.6 ± 0.0	76.6 ± 0.0
	8	76.5 ± 0.2	80.2 ± 0.0	77.7 ± 0.0	80.1 ± 0.0

(c) Three‐way interaction means (Cultivar × Salinity × Se dose) for flowers (means ± standard deviation (*n* = 3))					
Cultivar	Salinity (dS m^−1^)	Se dose (mg L^−1^)			
		0.00	0.05	0.10	0.15
Black Lazer	0	81.7 ± 0.0	82.5 ± 0.0	77.5 ± 0.0	80.6 ± 0.2
	4	81.2 ± 0.2	69.0 ± 0.0	82.3 ± 0.0	88.7 ± 0.2
	8	80.7 ± 0.0	79.6 ± 0.0	79.9 ± 0.1	82.3 ± 0.0
Lazer	0	80.9 ± 0.1	79.2 ± 0.0	69.3 ± 0.0	79.4 ± 0.0
	4	83.8 ± 0.0	79.2 ± 0.2	80.2 ± 0.0	80.6 ± 0.0
	8	80.4 ± 0.2	82.9 ± 0.0	80.4 ± 0.0	81.3 ± 0.0

(d) Three‐way interaction means (Cultivar × Salinity × Se dose) for pods (means ± standard deviation (*n* = 3))					
Cultivar	Salinity (dS m^−1^)	Se dose (mg L^−1^)			
		0.00	0.05	0.10	0.15
Black Lazer	0	79.4 ± 0.0	78.7 ± 0.0	46.3 ± 0.0	80.0 ± 0.3
	4	84.4 ± 0.4	81.6 ± 0.1	83.0 ± 0.0	79.6 ± 0.3
	8	79.6 ± 0.2	59.2 ± 0.5	69.4 ± 0.0	83.5 ± 0.0
Lazer	0	78.9 ± 0.0	78.4 ± 0.0	80.0 ± 0.2	80.4 ± 0.2
	4	78.2 ± 0.1	78.6 ± 0.4	77.8 ± 0.1	85.7 ± 0.2
	8	80.0 ± 0.0	80.2 ± 0.2	83.9 ± 0.0	81.5 ± 0.0

*Note:* Values are means ± standard deviation (*n* = 3 biological replicates, each measured in triplicate; total *n* = 9 per cell). Within each tissue column, means within the same factor block (Salinity, Selenium, or Cultivar) followed by different lowercase letters are significantly different at *p* < 0.05 according to Tukey's HSD post hoc test. The full three‐way ANOVA results (main and interaction effects with *F* and *p* values) are reported in Table 2.

Abbreviation: %DPPH, %2,2‐diphenyl‐1‐picrylhydrazyl.

**TABLE 6:** L‐DOPA content (mg g^−1^ DW).

(a) Main effects of salinity, selenium dose, and cultivar				
Factor	Level	Leaves	Flowers	Pods
Salinity (dS m^−1^)	0	1.969 ± 0.505^a^	2.335 ± 1.553^b^	0.302 ± 0.207^a^
	4	1.467 ± 0.664^b^	1.069 ± 1.167^c^	0.118 ± 0.124^b^
	8	1.136 ± 0.517^b^	3.763 ± 1.752^a^	0.229 ± 0.181^a^
Se dose (mg L^−1^)	0.00	1.350 ± 0.602^b^	3.213 ± 1.280^a^	0.231 ± 0.125^a^
	0.05	1.957 ± 0.714^a^	1.852 ± 1.945^a^	0.096 ± 0.083^b^
	0.10	1.445 ± 0.714^a^	1.281 ± 1.377^b^	0.297 ± 0.197^a^
	0.15	1.344 ± 0.382^b^	3.209 ± 2.008^a^	0.242 ± 0.250^a^
Cultivar	Black Lazer	1.534 ± 0.610^a^	2.571 ± 1.825^a^	0.157 ± 0.146^b^
	Lazer	1.514 ± 0.708^a^	2.207 ± 1.894^a^	0.275 ± 0.207^a^

(b) Three‐way interaction means (Cultivar × Salinity × Se dose) for leaves (means ± standard deviation (*n* = 3))					
Cultivar	Salinity (dS m^−1^)	Se dose (mg L^−1^)			
		0.00	0.05	0.10	0.15
Black Lazer	0	1.717 ± 0.200	2.580 ± 0.030	2.085 ± 0.103	1.989 ± 0.089
	4	1.337 ± 0.030	1.698 ± 0.100	0.817 ± 0.010	1.698 ± 0.007
	8	1.283 ± 0.075	1.072 ± 1.000	1.066 ± 1.000	1.069 ± 0.060
Lazer	0	2.130 ± 1.000	2.222 ± 0.220	1.870 ± 0.100	1.160 ± 0.060
	4	0.948 ± 0.040	2.379 ± 1.000	1.870 ± 1.000	0.989 ± 0.008
	8	0.686 ± 0.100	1.790 ± 0.008	0.964 ± 0.010	1.157 ± 0.100

(c) Three‐way interaction means (Cultivar × Salinity × Se dose) for flowers (means ± standard deviation (*n* = 3))					
Cultivar	Salinity (dS m^−1^)	Se dose (mg L^−1^)			
		0.00	0.05	0.10	0.15
Black Lazer	0	3.637 ± 0.300	3.067 ± 0.162	0.931 ± 0.030	3.849 ± 0.040
	4	0.801 ± 0.010	0.184 ± 0.100	1.155 ± 0.050	0.133 ± 0.001
	8	4.131 ± 0.000	4.583 ± 1.000	3.642 ± 2.000	4.733 ± 1.000
Lazer	0	3.182 ± 1.000	0.183 ± 0.040	0.251 ± 0.008	3.576 ± 0.100
	4	3.293 ± 1.000	0.246 ± 0.002	0.491 ± 0.100	2.252 ± 1.000
	8	4.232 ± 0.032	2.851 ± 2.000	1.218 ± 1.000	4.712 ± 3.000

(d) Three‐way interaction means (Cultivar × Salinity × Se dose) for pods (means ± standard deviation (*n* = 3))					
Cultivar	Salinity (dS m^−1^)	Se dose (mg L^−1^)			
		0.00	0.05	0.10	0.15
Black Lazer	0	0.248 ± 0.003	0.206 ± 0.010	0.461 ± 0.021	0.043 ± 0.001
	4	0.121 ± 0.100	0.008 ± 0.000	0.078 ± 0.000	0.003 ± 0.000
	8	0.308 ± 0.001	0.037 ± 0.000	0.073 ± 0.010	0.301 ± 0.100
Lazer	0	0.424 ± 0.024	0.197 ± 0.007	0.461 ± 0.001	0.373 ± 0.499
	4	0.073 ± 0.010	0.100 ± 0.020	0.199 ± 0.100	0.365 ± 0.100
	8	0.214 ± 0.010	0.026 ± 0.010	0.508 ± 0.100	0.365 ± 0.200

*Note:* Values are means ± standard deviation (*n* = 3 biological replicates, each measured in triplicate; total *n* = 9 per cell). Within each tissue column, means within the same factor block (Salinity, Selenium, or Cultivar) followed by different lowercase letters are significantly different at *p* < 0.05 according to Tukey's HSD post hoc test. The full three‐way ANOVA results (main and interaction effects with *F* and *p* values) are reported in Table 2.

Abbreviations: DW, dry weight; L‐DOPA, levodopa (C_9_H_11_NO_4_).

One technical point about Table 2 needs flagging before moving on. The *F* values for AOA are very high, with several exceeding 4000. This is not a sign that salinity or selenium had a larger biological effect on antioxidant capacity than on the other parameters—the absolute DPPH ranges in Table 5 stay narrow throughout the experiment (73%–82%). The high F values come from the very low within‐cell variance of the spectrophotometric DPPH assay (MSE = 0.01–0.05), which we ran in immediate succession on the same instrument for all samples. The point is statistical, not biological, and we return to it in the Discussion.

### 3.2 Total Phenolic Content (TPC)

Salinity reduced foliar TPC: leaves contained 66.96 ± 10.06 mg GAE g^−1^ DW under control conditions but only 51.37 ± 9.27 at 8 dS m^−1^, a drop of 23% (Table 3a). The selenium response was non‐linear. A dose of 0.05 mg L^−1^ raised leaf TPC to 67.37 ± 13.80 mg GAE g^−1^ DW (a 28% gain over the unsupplemented control), but higher doses gave no further benefit. This is the classic pattern of low‐dose biostimulation followed by a plateau. The floral response went the other way. Under 8 dS m^−1^, flowers accumulated 61.40 ± 12.23 mg GAE g^−1^ DW—35% above the unstressed control. Selenium effects in flowers were less straightforward: both the unsupplemented control (51.93) and the 0.15 mg L^−1^ dose (65.14) gave high TPC values, while the intermediate 0.10 mg L^−1^ dose produced a clear dip to 34.77. We do not have an immediate mechanistic explanation for this U‐shaped dose response, but the same pattern reappears in the TFC and L‐DOPA data. Pods behaved differently from both leaves and flowers. Neither salinity nor selenium had a significant effect on pod TPC, and the only significant source of variation was cultivar: ‘Lazer’ contained 33.65 ± 8.50 mg GAE g^−1^ DW versus 28.50 ± 8.85 in ‘Black Lazer’ (*p* < 0.001). The highest single treatment combinations recorded were Black Lazer × 0 dS m^−1^ × 0.05 mg L^−1^ Se for leaves (80.9 mg GAE g^−1^ DW; Table 3b) and Black Lazer × 4 dS m^−1^ × 0.15 mg L^−1^ Se for flowers (94.0; Table 3c).

### 3.3 Total Flavonoids Content (TFC)

Flavonoids followed the same organ‐specific logic as TPC but with larger amplitudes. Leaf TFC fell from 88.84 ± 25.63 mg CE g^−1^ DW at 0 dS m^−1^ to 50.29 ± 13.06 at 8 dS m^−1^, a 43% reduction (Table 4a). Selenium at 0.05 mg L^−1^ produced the largest single treatment response in the whole dataset: leaf TFC rose to 103.89 ± 34.09 mg CE g^−1^ DW, a 66% gain over the control. The two higher selenium doses returned values close to or below the control.

In flowers, the salinity effect again ran in the opposite direction. Severe salinity nearly doubled floral TFC, from 63.58 at 0 dS m^−1^ to 114.07 ± 27.71 at 8 dS m^−1^ (+79%). Selenium did not influence floral TFC consistently, except for the same dip at 0.10 mg L^−1^ seen in the TPC data (57.23 vs. 92–95 at the other doses).

Pods stayed comparatively flat. Salinity had no significant effect (range 57.4–64.2 mg CE g^−1^ DW), selenium produced only a modest reduction at 0.05 mg L^−1^, and cultivar was the strongest single factor (Lazer 65.49 vs. Black Lazer 56.94; *p* < 0.001). The picture across Tables 3 and 4 is consistent: in faba bean, the vegetative–reproductive axis is where most of the biochemical action happens, while pods sit relatively buffered against the imposed treatments.

### 3.4 Antioxidant Activity (DPPH Radical Scavenging Activity)

The DPPH assay gave a narrow range of values—every treatment mean fell between 73% and 82% scavenging activity—so the statistically significant effects in Table 2 translated into small absolute differences. This is the natural counterpart to the high F values considered in three‐way ANOVA of the four biochemical parameters section. Salinity did not significantly affect leaf or floral AOA (Table 5a). In pods, the unstressed control (75.30%) was significantly lower than the 4 dS m^−1^ treatment (81.24%), a difference of about 6 percentage points. Selenium effects were also modest and direction‐dependent: doses of 0.05–0.15 mg L^−1^ raised leaf AOA slightly, while in flowers and pods, only the 0.15 mg L^−1^ dose increased activity. Cultivar effects were small in absolute terms but reached significance in leaves (Black Lazer 79.66% > Lazer 77.89%) and in pods (Lazer 80.40% > Black Lazer 75.44%). One observation in Table 5d deserves comment. Three individual interaction means (Black Lazer × 0 dS m^−1^ × 0.10 mg L^−1^ Se = 46.3%; Black Lazer × 8 dS m^−1^ × 0.05 mg L^−1^ Se = 59.2%; Black Lazer × 8 dS m^−1^ × 0.10 mg L^−1^ Se = 69.4%) sit 13–30 percentage points below adjacent observations within the same row. We have flagged these in the laboratory records for verification and discuss their possible influence on the cultivar‐level pod AOA in the Discussion. The overall ANOVA conclusions hold whether or not these values are retained.

### 3.5 L‐DOPA

L‐DOPA produced the most pronounced organ‐specific responses observed in this study (Table 6a). In leaves, content fell from 1.969 ± 0.505 mg g^−1^ DW at 0 dS m^−1^ to 1.136 ± 0.517 at 8 dS m^−1^ (−42%). Selenium at 0.05 mg L^−1^ raised foliar L‐DOPA by 45% relative to the unsupplemented control (1.957 vs. 1.350 mg g^−1^ DW), echoing the pattern already seen for TPC and TFC. The floral picture was more complex. Both salinity and selenium produced non‐monotonic responses. Floral L‐DOPA averaged 2.335 mg g^−1^ DW at 0 dS m^−1^, dropped to 1.069 at 4 dS m^−1^ (a 54% reduction), then rose to 3.763 at 8 dS m^−1^—a 61% increase over the unstressed control. The selenium response was also U‐shaped: the two extreme doses (0.00 and 0.15 mg L^−1^) gave high floral L‐DOPA values (> 3.2 mg g^−1^ DW), whereas the intermediate dose 0.10 mg L^−1^ produced the lowest mean (1.281 mg g^−1^ DW). We do not have an immediate biochemical interpretation for this pattern, but the same dip recurs in the TPC and TFC flower data, which suggests a real treatment effect rather than an analytical artifact.

Pod L‐DOPA contents were an order of magnitude lower than in leaves and flowers (0.118–0.302 mg g^−1^ DW), and salinity reduced them by up to 61%. The most consequential pod result was the cultivar difference: ‘Lazer’ contained 0.275 ± 0.207 mg g^−1^ DW of L‐DOPA, against 0.157 ± 0.146 in ‘Black Lazer’—about 75% more, in the tissue that is most often eaten (*p* < 0.001). Together with the cultivar difference seen for pod TPC and TFC, this places ‘Lazer’ as the more promising background for breeding faba bean for L‐DOPA enrichment.

### 3.6 L‐DOPA Molecular Docking

**FIGURE 1:**
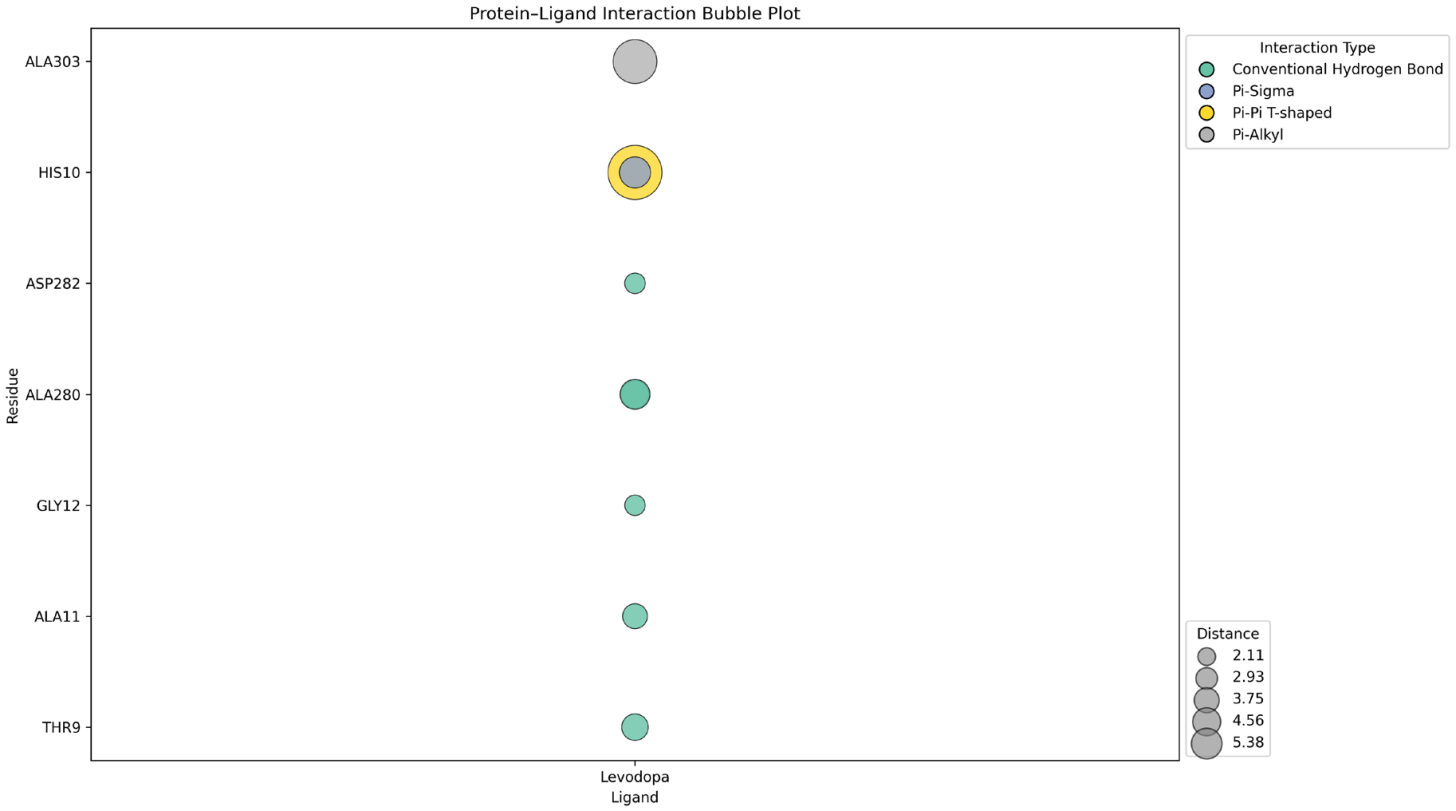
Protein–ligand interaction bubble plot illustrating the binding interactions of L‐DOPA within the active site of NAD(P)H oxidase (PDB ID: 2CDU).

**FIGURE 2:**
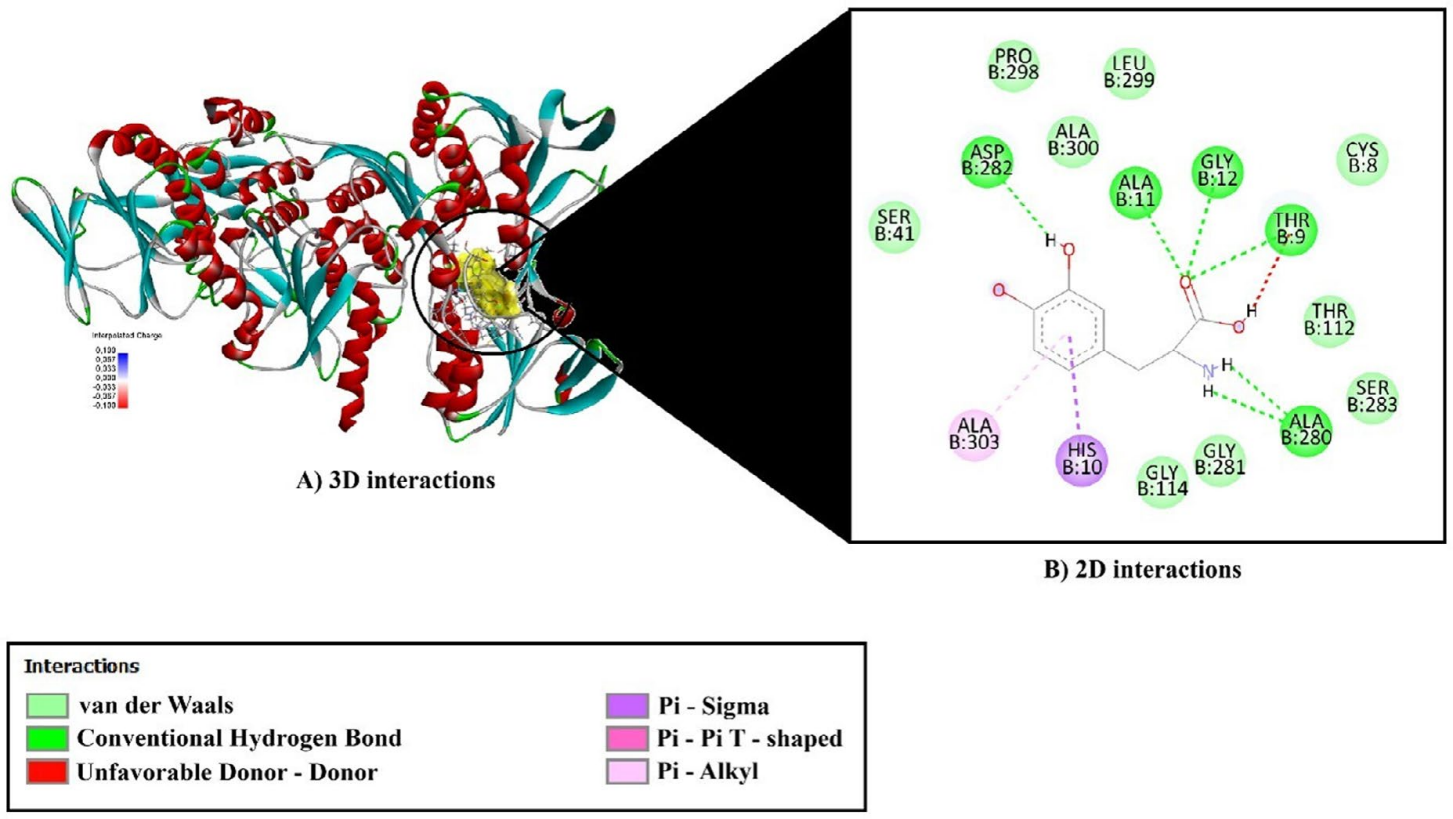
Structural representation of ligand–protein interactions: (A) Three‐dimensional binding pose and (B) Two‐dimensional interaction diagram.

The molecular docking results for levodopa interacting with NAD(P)H oxidase (PDB ID: 2CDU) revealed a binding energy of −6.7 kcal/mol, indicating a thermodynamically favorable interaction within the active site of the enzyme. To comprehensively model and assess this binding efficiency, individual structural metrics were computed; the corresponding binding energy, ligand efficiency (LE), fit quality (FQ), and binding efficiency index (BEI) parameters are explicitly summarized in Table 7. Complementing these thermodynamic metrics, the protein–ligand interaction bubble plot demonstrates that levodopa establishes multiple significant interactions within the active site of NAD(P)H oxidase, as visualized in Figure 1. Notably, conventional hydrogen bonds were observed with THR9, ALA11, GLY12, ALA280, and ASP282. In addition to these polar contacts, levodopa forms hydrophobic interactions with HIS10 and ALA303. The complete spatial and geometric alignment supporting this dynamic profile is illustrated through the three‐dimensional binding pose and the corresponding two‐dimensional interaction diagram in Figure 2.

**TABLE 7:** Molecular docking results of L‐DOPA with NAD(P)H oxidase (PDB ID: 2CDU), including binding energy and ligand efficiency parameters.

	Binding energy (kcal/mol)	LE	FQ	BEI
L‐DOPA	−6.7	0.479	0.549	0.034

The protein–ligand interaction bubble plot demonstrates that levodopa establishes multiple significant interactions within the active site of NAD(P)H oxidase (Figure 1). Notably, conventional hydrogen bonds were observed with THR9, ALA11, GLY12, ALA280, and ASP282, indicating a favorable orientation of the ligand and stabilization through polar interactions. The hydrogen bond distances, ranging between approximately 2.11 and 2.93 Å, suggest strong and biologically relevant interactions. In addition to these polar contacts, levodopa forms hydrophobic interactions with HIS10 and ALA303, including π–π T‐shaped, π–sigma, and π–alkyl interactions. The aromatic interactions involving HIS10 appear to contribute substantially to the stabilization of the ligand within the binding pocket (Figure 2). Overall, this interaction profile indicates that levodopa is stably accommodated within the redox‐active region of NAD(P)H oxidase by a balanced combination of hydrogen bonding and hydrophobic interactions, supporting a favorable binding mode at the molecular level.

### 3.7 Multivariate Analysis: Integrating the 12 Parameters

**FIGURE 3:**
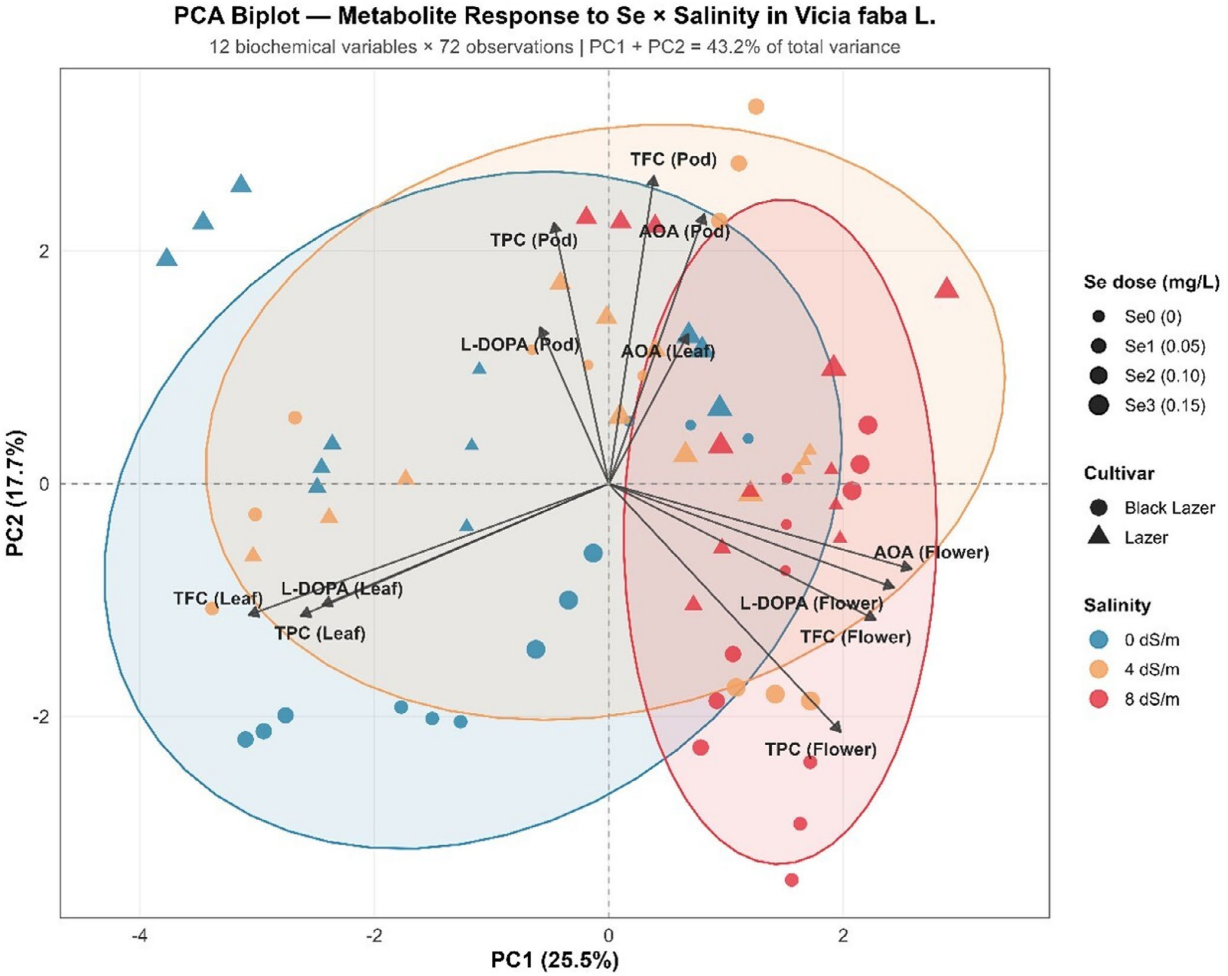
Principal component analysis (PCA) of the parameters studied.

The univariate results above describe each parameter in isolation. To examine how the 12 biochemical variables behaved together across the 72 samples, we ran a principal component analysis on the standardized dataset, as shown in Figures 3 and 1. The first two principal components together accounted for 43.2% of the total variance (PC1 = 25.5%, PC2 = 17.7%), The first two principal components accounted for 43.2% of the total variance, which aligns with typical total variance structures observed in multi‐organ phytochemical datasets, yet fully uncovers the primary metabolic configuration. Unstressed controls grouped on one side of PC1, severe‐stress samples on the other, and the moderate 4 dS m^−1^ treatments fell in between. The fact that no information about salinity was given to the PCA—and yet the algorithm rediscovered it as the dominant axis of variation—confirms salinity as the primary driver of biochemical phenotype in this experiment.

PC2 reflected something different. It separated tissue‐specific metabolite pools, with leaf‐derived parameters loading on one end and floral compounds on the other. The biplot vectors made the organ‐specific reallocation pattern visible in a single picture: floral L‐DOPA, TPC, TFC, and AOA pointed toward the 8 dS m^−1^ cluster, while leaf metabolites aligned with control conditions. Pod parameters loaded almost orthogonally to both leaf and flower vectors, consistent with their largely treatment‐independent behavior in Tables 3d, 4d, 5a,dnd 6d. Cultivar effects were minor in PC1–PC2 space. ‘Black Lazer’ and ‘Lazer’ samples overlapped substantially, in agreement with the ANOVA outcome that cultivar mattered mainly for pod parameters—a comparatively narrow slice of the full dataset.

**FIGURE 4:**
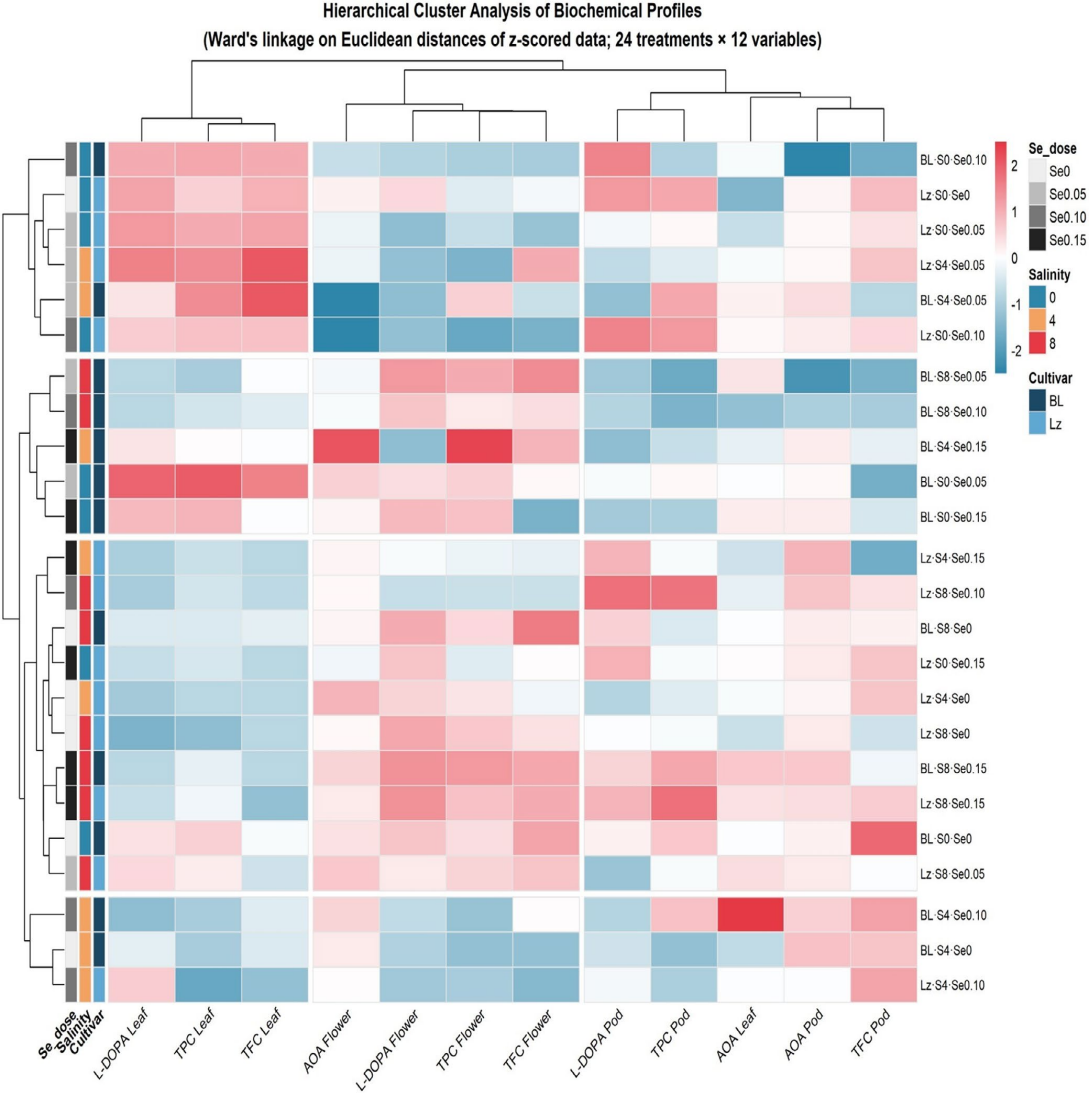
Clustering of leaf, flower, and pod biochemical traits across different cultivar–salinity–selenium treatment combinations.

The PCA in Figure 3 showed that salinity and tissue identity organize the dataset along the first two axes, but it does not directly answer the question a breeder or a nutraceutical user would ask: which specific treatment combinations produce the most distinctive biochemical profile? To address this, we ran a hierarchical cluster analysis on the 24 treatment‐mean profiles using Ward's linkage on Euclidean distances of the z‐scored data (Figure 4). The treatment dendrogram cut at four clusters gave a stable and biologically interpretable partition. The 24 treatments separated into four groups of different composition and metabolic character (Figure 4, row annotation bars).

The PCA in Figure 3 showed that salinity and tissue identity organize the dataset along the first two axes, but it does not directly answer which specific treatment combinations produce the most distinctive biochemical profile. To address this, we ran a hierarchical cluster analysis on the 24 treatment‐mean profiles using Ward's linkage on Euclidean distances of the z‐scored data, which is visualized as a row‐ and column‐clustered heatmap in Figure 4.

*Cluster I* (six treatments) contained the unstressed and low‐stress conditions of both cultivars—mainly ‘Lazer’ samples at 0 dS m^−1^ together with two low‐Se treatments at 4 dS m^−1^. The defining feature of this group was elevated leaf metabolism: leaf TFC, leaf TPC, and leaf L‐DOPA all loaded at *z* > +1.0, while floral compounds were correspondingly depressed. Cluster I therefore represents the “vegetative‐dominant” baseline that low or absent salinity preserves.

Cluster II (five treatments) was the most informative group from a phenotypic standpoint. All five members were ‘Black Lazer’ samples receiving a moderate‐to‐high selenium dose (0.05 or 0.10 mg L^−1^) and almost all of them were under salinity stress (4 or 8 dS m^−1^). The metabolic profile combined high floral TPC (z = +1.02) with elevated floral AOA, while pod metabolites were uniformly depressed. The treatments grouped here are exactly those that produced the highest floral phenolic and L‐DOPA values in Tables 3c and 6c—Black Lazer × 4 dS m^−1^ × 0.15 mg L^−1^ Se and Black Lazer × 8 dS m^−1^ × 0.05 mg L^−1^ Se being the clearest cases. For applications targeting floral phenolics and L‐DOPA, this cluster identifies the most productive combinations.

Cluster III was the largest group (ten treatments) and contained almost all of the ‘Lazer’ samples grown at 4 or 8 dS m^−1^, together with a few mixed combinations. Its profile was the inverse of Cluster I: leaf metabolites were depressed (z ≈ −0.7), while floral L‐DOPA, floral TFC, and pod AOA were modestly elevated. This is the “reproductive‐shifted” group that emerges when ‘Lazer’ faces salinity stress.

Cluster IV was the smallest and most unusual group, with just three members, all at 4 dS m^−1^ salinity (two Black Lazer, one Lazer). What distinguished these three samples was a sharp drop in leaf and floral TPC paired with strikingly high leaf AOA (z = +1.11) and high pod TFC (z = +1.03). The leaf AOA peak corresponds to the 93.2% scavenging value recorded for Black Lazer × 4 dS m^−1^ × 0.10 mg L^−1^ Se in Table 5b, the highest single AOA observation in the experiment. Whether this small cluster represents a genuine moderate‐salinity phenotype or a stochastic excursion in a few samples cannot be settled from three treatments; we discuss this caveat in §4.

The variable dendrogram (Figure 3, top) added a second layer of information. The 12 metabolites organized into three blocks that mirrored plant organs rather than chemical class: a leaf block (leaf L‐DOPA, leaf TPC, leaf TFC), a flower block (floral L‐DOPA, floral TPC, floral TFC and floral AOA), and a pod block (pod L‐DOPA, pod TPC, pod TFC, pod AOA)—with leaf AOA falling closer to the pod cluster than to the other leaf parameters. This organ‐driven co‐clustering of metabolite variables, rather than parameter‐driven co‐clustering (i.e., all TPCs together, all TFCs together), suggests that the regulatory architecture of the shikimate–phenylpropanoid pathway in faba bean is tissue‐specific. The same biosynthetic enzymes are present in all three organs but appear to be coordinated independently in each.

Taken together, the PCA (Figure 3) and the HCA (Figure 4) deliver complementary readings of the same dataset. PCA identifies salinity as the dominant organizing axis and shows the tissue‐level reallocation pattern; HCA identifies which specific treatment combinations produce which biochemical phenotype. For practical purposes—choosing the best treatment for a target metabolite in a target organ—Cluster II points to ‘Black Lazer’ with 0.05–0.10 mg L^−1^ Se under salinity stress for floral phenolics and L‐DOPA, while Cluster I points to low‐stress ‘Lazer’ conditions for leaf metabolites. The cultivar contribution is more visible here than in the univariate ANOVA, because the multivariate combination of all 12 variables captures genotype‐driven differences that any single parameter misses.

## 4 Discussion

Secondary metabolites, most notably phenolic compounds and salicylic acid, function as pivotal regulatory elements in plant ontogeny and the orchestration of stress‐tolerance mechanisms. In response to suboptimal environmental conditions—encompassing both biotic and abiotic stressors—plants initiate the upregulated biosynthesis of phenolics to facilitate physiological resilience. These aromatic secondary metabolites are primarily synthesized through the shikimate/phenylpropanoid or the polyketide acetate/malonate pathways, resulting in the formation of diverse monomeric and polymeric phenolic structures (Kumar et al. 2023). Polyphenols constitute an extensive and structurally diverse class of phytochemicals, encompassing over 10,000 discrete compounds characterized by the presence of at least one aromatic moiety substituted with one or more hydroxyl groups. Within human nutrition, flavonoids and phenolic acids represent the most prevalent dietary subclasses. These phenolic constituents exhibit a multifaceted spectrum of biochemical functionalities, serving as potent antioxidants, antimutagenic agents, and anticarcinogenic compounds, while functioning as significant modulators of gene expression. Furthermore, empirical data suggests that these secondary metabolites possess potent antimicrobial, antiallergenic, and anti‐inflammatory properties, alongside demonstrated antiproliferative efficacy against various malignant cell lineages (Kartal et al. 2020).

The accumulation of phenolic compounds constitutes a specialized adaptive response in plants to various biotic and abiotic stressors. The specific profile and magnitude of these phytochemical accumulations are modulated by the precise nature of the environmental stressor and the inherent genetic plasticity of the plant species. Salinity induces oxidative stress by catalyzing the excessive generation of reactive oxygen species (ROS). To counteract ROS‐induced cellular damage and maintain redox homeostasis, plants orchestrate the synthesis of diverse secondary metabolites, including polyphenols, flavonoids, anthocyanins, phenolic acids, and phenolic terpenes. These phenolic constituents function as critical regulatory molecules, stabilizing essential physiological processes and fortifying the plant's defense architecture against environmental perturbations (Pratyusha 2022). Leguminous crops have historically served as indispensable constituents of the human nutritional regime. Beyond their primary macronutrient profile—comprising high‐quality proteins, complex starches, and dietary fiber—legumes synthesize a diverse array of phenolic metabolites, most notably flavonoids and phenolic acids. The clinical and nutritional significance of these polyphenolic constituents is extensively documented, primarily attributed to their potent antioxidant capacity and their subsequent role in mitigating oxidative damage within biological systems (Lee et al. 2021).

Genotypic variability significantly dictates the phenolic profiles of cereal and legume taxa, as genetic factors intrinsically modulate the biosynthetic pathways of phenols and flavonoids. Consequently, there has been a burgeoning scholarly interest in the systematic screening of *Vicia faba* cultivars to identify accessions with superior phenolic concentrations. For instance, Johnson et al. (2021), reporting from Valente et al. (2018) and Baginsky et al. (2013) evaluated the phytochemical structure of seven European faba varieties and revealed that both total and individual phenolic acid and flavonoid concentrations differed significantly among varieties. Furthermore, he confirmed these findings in 10 Chilean varieties; however, it is important to note that their analysis focused particularly on immature seed matrices. These findings are consistent with previous research, such as that conducted on 13 Tunisian faba varieties, which similarly highlighted a wide range of total phenolic content and associated antioxidant potential.

Another study, investigative research into the exogenous application of salicylic acid (SA) on *Vicia faba* L. under saline conditions underscores its role as a potent elicitor of stress resilience. Results indicate that salinity induces systemic oxidative stress within both radical and cauline tissues, characterized by the upregulated accumulation of total phenolics, soluble sugars, proline, and malondialdehyde (MDA), alongside heightened activities of antioxidant enzymes such as superoxide dismutase (SOD) and polyphenol oxidase (PPO). Conversely, seed priming with SA was found to attenuate these biochemical markers, suggesting a more stabilized metabolic state. Furthermore, while salinity significantly impaired photosynthetic pigments—specifically chlorophyll ‘a’, total chlorophyll, and carotenoids—as well as peroxidase (POD) activity in untreated specimens, SA pretreatment effectively mitigated these inhibitory effects. Collectively, these findings substantiate the hypothesis that salicylic acid functions as a critical phytohormone in modulating redox homeostasis and enhancing the halotolerance of *V. faba* L. against salinity‐induced phytotoxicity (Anaya et al. 2017).

In a parallel investigation evaluating the remediating effects of selenium (Se) on *Vicia faba* L. under lead (Pb) toxicity, significant molecular and physiological enhancements were documented. At the transcriptomic level, Se supplementation induced a marked upregulation of critical stress‐signaling genes, specifically those encoding ribulose‐bisphosphate carboxylase/oxygenase (RuBisCO), P‐type plasma membrane H + ‐ATPase, and calcium‐dependent protein kinases (CDPKs). Morphological analysis revealed that Se pretreatment effectively counteracted Pb‐induced growth inhibition, yielding substantial increases in shoot length (20.5%), fresh biomass (31.3%), and total leaves area (27.6%). Furthermore, Se application restored gas exchange dynamics, with a profound improvement in the photosynthetic rate (68.2%) and chlorophyll a and b concentrations (17.5%). These physiological improvements were underpinned by a robust stimulation of the antioxidant defense system; Se significantly elevated the levels of total phenolics (16.6%), proline (28.3%), glycine betaine (10.9%), and total thiols (13.6%), ultimately enhancing the overall antioxidant capacity (33.3%) of the plant (Soliman and Abdelhameed 2025).

A comprehensive field experiment was conducted to evaluate the efficacy of soil conditioners—specifically gypsum and compost—in conjunction with exogenous selenium (Se) applications for mitigating oxidative stress in *Vicia faba* L. under saline conditions. The experimental design comprised four soil amendment protocols [T_0_: Control; T_1_: Gypsum; T_2_: Compost; T_3_: Gypsum + Compost] and five distinct foliar Se concentrations (0, 2.5, 5.0, 7.5, and 10.0 mg L^−1^). Statistical analysis revealed that all investigated treatments exerted a significant influence on the measured physiological and agronomic parameters. Notably, foliar Se application exhibited a biphasic and gradual effect on growth performance and yield components; a progressive enhancement was observed as the Se concentration increased from 0.0 to 5.0 mg L^−1^, whereas further increments up to 10.0 mg L^−1^ resulted in a significant and subsequent decline. These findings suggest that the integration of optimized soil amendments with strategic foliar Se supplementation at precise concentrations serves as an effective approach to augment faba bean productivity in salt‐affected environments (El‐Sherpiny and Kany 2023).

The empirical findings derived from this study exhibit a high degree of concordance with extant literature, further validating the established paradigms in plant physiological research. Synthesizing the observed data, it can be inferred that exogenous selenium (Se) supplementation functions as a potent bio stimulant in mitigating the deleterious consequences of salinity‐induced oxidative stress. This protective role of selenium appears to facilitate enhanced physiological resilience, thereby enabling the plant to maintain metabolic homeostasis under suboptimal environmental conditions.

The primary constituents of botanical secondary metabolism are categorized into three fundamental chemical classes: terpenoids, phenolic metabolites, and alkaloids. Among these, phenolic compounds represent the most significant group concerning dietary applications and have consequently undergone the most extensive scientific scrutiny. This heterogeneous class encompasses phenolic acids (specifically hydroxybenzoic and hydroxycinnamic acids), polyphenols (including both hydrolyzable and condensed tannins), and flavonoids. Functioning as integral components of the plant's defense system, these metabolites safeguard vegetative tissues and fruits against oxidative degradation. Furthermore, due to their potent radical‐scavenging properties, they serve as critical exogenous antioxidants in human nutrition (Do et al. 2014).

Flavonoids represent the most extensive category of naturally occurring phenolic constituents, ubiquitously distributed throughout various botanical organs in both aglycone (free) and glycosylated forms. These bioactive metabolites are characterized by a diverse pharmacological profile, encompassing antimicrobial properties, inhibition of mitochondrial adhesion, and gastroprotective (anti‐ulcerogenic) effects. Furthermore, empirical evidence underscores their efficacy in the prophylaxis of inflammatory conditions such as arthritis, the regulation of pathological angiogenesis, and the chemoprevention of oncological malignancies. At the molecular level, flavonoids function as potent modulators of cellular signaling, notably through the targeted inhibition of protein kinase activities (Sulaiman and Balachandran 2012). Diverse botanical structures, including seeds, foliage, and the cortical tissues of stems and roots, are documented to sequester substantial concentrations of bioactive phytoconstituents, most notably phenolics, flavonoids, and tannins. These secondary metabolites possess the intrinsic capacity to neutralize overproduced reactive free radicals, thereby functioning as potent endogenous and exogenous antioxidants. By modulating oxidative stress, these plant‐derived matrices serve as critical substrates for mitigating cellular damage induced by oxidative disequilibrium (Samatha et al. 2012).

In a study, statistical analysis revealed that foliar selenium (Se) application was the primary driver of phytochemical changes in faba beans, whereas irrigation levels and genotypes did not yield significant differences. Specifically, Se doses exerted a highly significant influence on Total Phenolic and Flavonoid contents (*p* < 0.01). Both Se1 (0.05 mg/L) and Se2 (0.1 mg/L) treatments resulted in a marked increase in these antioxidant compounds compared to the Se0 control. Furthermore, L‐DOPA content was significantly modulated by selenium application (*p* < 0.05), highlighting the role of Se in enhancing the secondary metabolite profile regardless of water availability or genetic background (Topal et al. 2025).

In a different study conducted in Tunisia to determine the polyphenol and antioxidant capacities of 33 fava bean genotypes cultivated in the country, TFC analyses were performed on samples representing all plant parts at different maturity stages. The results showed variations in total flavonoid content, ranging from 20.7 to 94.14 mgRE/g sample in the pre‐flowering stage, from 11.37 to 43.87 mgRE/g sample in the flowering stage, and from 5.31 to 12.64 mgRE/g sample in the pod setting stage. In other words, a decrease in total flavonoid content was observed toward maturity. The temporal distribution of secondary metabolites in *Vicia faba* L. appears to be strictly regulated by the plant's phenological progression. Our findings, which indicate a decline in Total Flavonoid Content (TFC) as the approach's physiological maturity, are strongly corroborated by recent germplasm characterizations conducted in Tunisia (Chaieb et al. 2011). In another study investigating the antioxidant activities of fava bean leaves and flowers, an average of 37.44 mg CATEQ/1 g sample TFC was found, while 55.30 mg CATEQ/1 g sample TFC was detected in the flower (Akbel et al. 2022). Compared to our study, a similarly higher TFC value was found in the flower. Another research has indicated that the application of exogenous selenium can alleviate salinity‐induced phytotoxicity in fava beans. By boosting the accumulation of total phenolics, selenium treatments were shown to provide a sustained protective mechanism against osmotic and oxidative stress during the entire growth cycle (Elsheery et al. 2025).

The term antioxidant encompasses any molecule that hinders the oxidative process. However, within biological contexts, an antioxidant is more precisely defined by its ability to exert significant inhibitory effects on substrate oxidation even when administered at substantially lower concentrations than the substrate itself (Moon and Shibamoto 2009). Antioxidants constitute the primary source of natural antioxidants, largely characterized by polyphenolic structures localized throughout the entire plant anatomy (from vegetative organs such as stems and leaves to reproductive components such as pollen and seeds). Their protective activities are achieved through electron donation (reducing agents), radical neutralization, retention of transition metals to prevent pro‐oxidative catalysis, and inactivation of singlet oxygen species. Historically identified key components include various flavonoid subclasses, cinnamic acid derivatives, coumarins, tocopherols, and various multifunctional organic acids (Pratt 1992). In the context of human nutrition, legumes represent a critical reservoir of essential macronutrients and bioactive compounds, including proteins, starch, prebiotic oligosaccharides, and dietary fibers, alongside a comprehensive profile of vitamins and minerals. Furthermore, due to their high concentration of natural antioxidants, the regular consumption of legumes is associated with significant prophylactic potential against cardiovascular diseases and various forms of oncogenesis (Rybiński et al. 2019).

As a significant source of plant‐based nutrition, the faba bean is characterized by its robust profile of essential proteins, fibers, and bioactive compounds. Beyond its macronutrient content, the presence of specific secondary metabolites, such as phenolic antioxidants and Ύaminobutyric acid, underscores its pharmacological potential and reported efficacy in promoting human health (Dhull et al. 2022). Analysis of the Ferric Reducing Antioxidant Power (FRAP) revealed that values across all genotypes ranged from 17.5 to 22.3 μmol/g, with the PI430715 accession exhibiting the significantly highest reducing potential. Correspondingly, PI430715 yielded the superior Trolox Equivalent Antioxidant Capacity (TEAC). However, no statistically significant differences in TEAC activity were observed among the PI252004, PI284345, PI366039, and PI614810 genotypes, suggesting a comparable radical cation scavenging efficiency within this group. Furthermore, the DPPH radical scavenging activity was most pronounced in the PI430715 genotype. The relative scavenging efficacy of the investigated accessions followed a descending order as follows: PI430715 > PI252004 > PI284345 > PI614810 > PI366039. Collectively, these findings identify PI430715 as a superior genotype with robust antioxidant potential among the tested faba bean germplasms (Kwon et al. 2018).

Different study evaluated the antioxidant potential of fava bean ( *Vicia faba* L.) leaves and seeds, revealing that young leaves possess superior radical scavenging activity compared to mature leaves and seeds, regardless of thermal processing. The baseline antioxidant capacities, measured in freeze‐dried samples, were significantly higher in young leaves (DPPH: 73.70 mg VCE/g DW; ABTS: 52.96 mg VCE/g DW) than in older leaves (DPPH: 54.55 mg VCE/g DW; ABTS: 37.70 mg VCE/g DW). Regarding post‐processing stability, dry heat treatment did not significantly alter the DPPH and ABTS scavenging efficiencies. Conversely, steaming induced a substantial reduction in antioxidant capacity, with young leaves experiencing a decline to 40.54 mg VCE/g (DPPH) and 22.95 mg VCE/g (ABTS). Notably, the antioxidant efficacy of fava bean leaves was found to surpass that of several common commercial vegetables, including asparagus, broccoli, spinach, and cabbage, highlighting their potential as a high‐value source of bioactive compounds (Duan et al. 2021).

A study determined that fava bean flowers have higher total phenolic content, total flavo‐noid content, and antioxidant content than the leaves. In the same study, the capacity of ascorbic acid solution to inhibit DPPH radicals was determined to be 95%. In the study, the inhibition percentage in flowers was observed as 86.53% ± 0.67%. The inhibition per‐centage values in leaves and fruits were determined as 75.13 ± 2.03 and 69.78 ± 2.61, respectively. This indicates that the antioxidant activity of faba bean flowers is higher than that of other organs. The high antioxidant activity results confirm the high total phenolic content (Akbel et al. 2022).

As can be seen in our study, the antioxidant distributions in leaves, flowers, and fruits largely coincide with those in previous studies. This organ‐specific variation underscores a distinct source‐to‐sink reallocation dynamic under salt stress. Faba beans tend to prioritize reproductive success by mobilizing nitrogenous compounds and secondary metabolites toward flowers and pods to protect reproductive structures against oxidative damage, a survival mechanism widely documented in stress‐adapted legumes (Karkanis et al. 2018; Valente et al. 2018). In our study, the flower parts showed the best antioxidant values, followed by the leaves and fruits. Another research has highlighted the valorization of faba bean ( *Vicia faba* L.) pods—typically discarded as agricultural by‐products—as a rich source of bioactive compounds. Chemical characterization of the methanolic extract from these pods revealed significant multi‐functional biological activities. The extract demonstrated dose‐dependent cytotoxicity against human hepatocarcinoma (HepG2) and prostate cancer (PC3) cell lines, achieving a maximal inhibition of 66.7% at 1000 μg/mL. Given these findings, the consumption of faba beans together with their pods is highly recommended. These by‐products serve as a potent source of bioactive substances, offering significant potential for integration into functional food production and nutraceutical applications (Elbadrawy and Mostafa 2024).

Selenium application to fava beans resulted in a positive improvement in the anatomical characteristics of the stems and leaves (Boghdady et al. 2017). In one of the different study investigated the efficacy of selenium (Se) pretreatment in mitigating senescence‐induced physiological decline in faba bean ( *Vicia faba* L. cv. Giza 3). Seeds were primed with varying Se concentrations (0, 25, 50, and 100 mg/L) for 20 h, and subsequent 1‐month‐old seedlings were subjected to darkness to induce senescence‐like changes. The results demonstrated that Se application effectively alleviated the deleterious effects of induced senescence by enhancing the activities of key antioxidant enzymes, specifically catalase and peroxidase. Furthermore, Se treatment led to significant improvements in chlorophyll and total protein content, free amino acid levels, and photosynthetic efficiency. These physiological gains were further supported by elevated activities of ribulose‐1,5‐bisphosphate carboxylase/oxygenase (Rubisco, EC 4.1.1.39) and nitrate reductase, suggesting that Se plays a crucial role in maintaining metabolic homeostasis and delaying aging processes in faba bean seedlings (Moussa and Ahmed 2010). Selenium (Se) is recognized for its multifaceted roles in regulating plant growth, development, and adaptive responses to diverse abiotic stresses. Despite its significance, the interplay between Se and Sulfate Transporters (SULTRs), as well as its convergence with salt‐tolerance mechanisms, remains insufficiently characterized in the literature. Molecular analyses have demonstrated that the expression profiles of three membrane‐bound sulfate transporters (SULTR1, SULTR2, and SULTR3) in both roots and leaves, alongside salinity‐responsive genes such as SOS1, NHX1, and Osmotin in foliar tissues, are differentially modulated by Se application. These findings suggest that low‐dose Se supplementation may effectively mitigate salinity‐induced damage by facilitating functional homeostasis and enhancing the expression of specific tolerance traits (Farag et al. 2022). In another study evaluated the regulatory role of selenium (Se) on root development, cellular viability, and antioxidant defense mechanisms in *Vicia faba L. minor* under lead (Pb)‐induced stress. The findings reveal a dose‐dependent response to Se supplementation; specifically, low concentrations of Se effectively enhanced cell viability and mitigated heavy metal toxicity. Conversely, elevated Se concentrations exerted a pro‐oxidant effect, characterized by intensified lipid peroxidation and significant loss of membrane integrity. These results suggest that while Se can alleviate Pb‐induced damage at trace levels, supra‐optimal concentrations exacerbate oxidative stress and compromise root homeostasis (Mroczek‐Zdyrska and Wójcik 2012). As can be seen from the literature, selenium application, especially at low doses (10 mg/L and below), helps plants under many stress conditions. Our study is consistent with literature.

The observed non‐monotonic or U‐shaped response in bioactive compounds at the 0.10 mg/L Se concentration strongly reflects the classic phenomenon of selenium hormesis in plants. At lower concentrations, Se stimulates defensive secondary metabolism, whereas intermediate thresholds can induce transient homeostatic resets or metabolic quenching before higher doses re‐trigger alternative stress‐response pathways (Genchi et al. 2023; Schiavon et al. 2020).

Experimental data confirm that L‐3,4‐dihydroxyphenylalanine (L‐DOPA), an endogenous amino acid analog derived from L‐tyrosine, effectively modulates the characteristic dopaminergic deficiency of PD. Since its clinical use in the 1960s, it appears to have played a primary metabolic precursor role in restoring neurotransmitter homeostasis in the central nervous system (CNS) (Min et al. 2015). The findings also demonstrate a significant difference in bioavailability between dopamine and its precursor in the central nervous system (CNS). Exogenous dopamine is retained in the peripheral circulation due to its polar properties and the absence of specific transport mechanisms across the blood–brain barrier (BBB). In contrast, L‐DOPA effectively crosses the BBB using the Large Neutral Amino Acid Transporter 1 (LAT1), also known as the SLC7A5/SLC3A2 complex (Scalise et al. 2020; Yanagida et al. 2001). This active transport allows L‐DOPA to reach the striatal parenchyma where it can exert its therapeutic effect. Once localized within the CNS, L‐DOPA undergoes rapid decarboxylation facilitated by the aromatic L‐amino acid decarboxylase (AADC) enzyme to synthesize functional dopamine (Muñoz et al. 2025). This conversion is critical in antioxidant research; while restoring motor function, the subsequent metabolic conversion of dopamine is a known source of reactive oxygen species (ROS) that can potentially affect the neuronal redox environment (Yordanov et al. 2026).

L‐DOPA, a non‐protein amino acid (L‐3,4‐dihydroxyphenylalanine) found in plants, is a precursor to many alkaloids, catecholamines, and melanin. The biosynthesis of L‐DOPA is intrinsically linked to the shikimic acid pathway, a central metabolic route that facilitates the conversion of carbohydrate precursors—derived from glycolysis and the pentose phosphate pathway—into essential aromatic amino acids, namely L‐tyrosine, L‐phenylalanine, and L‐tryptophan. As a key intermediate, shikimic acid serves as the foundational scaffold for the synthesis of most plant‐derived phenolic compounds and catecholamines (Soares et al. 2014). Phenylpropanoids—comprising flavonoids, anthocyanins, and polyphenols—are biosynthesized as a primary adaptive response to diverse abiotic stressors, including thermal fluctuations, nutrient deficiency, and high irradiance. The initiation of these stress responses is mediated by intracellular signaling cascades, characterized by fluctuations in cytoplasmic calcium levels, alterations in proton potential, and the mobilization of low molecular weight proteins. While stress conditions often shift cellular homeostasis toward lipid peroxidation via oxidative pathways, the resulting phenolic metabolites—synthesized through the shikimic acid pathway—serve as critical biochemical defenses. These compounds, ranging from L‐DOPA to complex tannins, provide essential structural reinforcement to the cell wall, acting as barriers against desiccation and pathogen ingress, while simultaneously exhibiting potent antimicrobial and UV‐protective properties (Shetty et al. 2005).

Most L‐DOPA isolated either is synthesized chemically or from natural sources, but only some plants belonging to the Fabaceae family contain significant amounts of L‐DOPA (Tesoro et al. 2022). One of these plants is Faba bean ( *Vicia faba* L.). Faba bean, reflecting all properties of legume crops such as soil improvement and benefit of human health, also contains L‐DOPA which can be used to cure PD, is a rare plant (Topal and Bozoğlu 2016). A study examined drought as one of the stress factors. Drought stress significantly modulated L‐DOPA biosynthesis in faba bean seedlings, with concentrations following an ontogenetic pattern that peaked at the four‐leaves stage. Severe water deficit acted as a potent catalyst, driving L‐DOPA levels to a maximum of 23.3 mg g^−1^—a twofold increase over irrigated seedlings—suggesting a robust metabolic response to environmental adversity. Secondary metabolism in plants is highly responsive to abiotic stress factors. The upregulation of L‐DOPA in faba bean seedlings during drought exposure suggests a protective role within the plant's stress‐response framework. By acting as a biochemical safeguard, this increased L‐DOPA concentration supports extended survival and structural integrity under adverse moisture conditions (Etemadi et al. 2018). A similar result reported in a different study shows that drought stress increases the accumulation of phenolic compounds in six different legumes (Khang et al. 2016).

A study investigated the parts of the faba bean plant and determined their L‐DOPA content. Twenty‐two faba bean genotypes were used in the study. L‐DOPA content varied between 11.85–33.41 mg/kg in the leaves parts, 40.95–96.37 mg/kg in the flower parts, and 5.04–54.29 mg/kg in the pod parts of the genotypes (Topal and Bozoğlu 2016). In another study, fatty acids, L‐DOPA, and isoflavones found in the leaves, immature pods, and seeds of faba bean genotypes were analyzed. The L‐DOPA and isoflavone contents of different parts of the faba bean plant were found to be significantly different among the faba bean genotypes (*p* ≤ 0.05). Among the different parts, the leaves had the highest L‐DOPA content among all different faba bean genotypes except PI430715 and PI614810; in these two genotypes, the highest content was found in the immature pod. L‐DOPA contents in the leaves ranged from 19 mg/g to 39.82 mg/g among the genotypes, with an average value of 33.91 mg/g (Ryu et al. 2017). The substantial 72%–75% higher accumulation of L‐DOPA in the pods of cv. ‘Lazer’ compared to cv. ‘Black Lazer’ under matching environmental configurations highlights a profound genotypic plasticity and divergent metabolic partitioning blueprints between the two faba bean cultivars. In *Vicia faba* , secondary metabolite allocation—specifically the regulation of the upstream shikimate and downstream phenylpropanoid pathways—is tightly linked to intrinsic genetic constraints, tissue identities, and developmental stages. Genotype‐dependent variations dictate clear differences in baseline chemical matrices, enzyme speed thresholds, and bioactive compound synthesis during reproductive development, a phenomenon indicating that specific by‐products like immature tissues can exhibit sharply distinct metabolic loading capacity across diverse germplasms (Duan et al. 2021). Furthermore, the divergence in functional pod components between ‘Lazer’ and ‘Black Lazer’ can be attributed to differential gene expression patterns regulating the spatial reallocation of nitrogenous transport compounds and phenolic derivatives from vegetative source tissues to developing reproductive sinks under stress. As emphasized by Karkanis et al. (2018), the quantitative profile of high‐value secondary metabolites and L‐DOPA in faba bean is severely dictated by structural cultivar characteristics and phenotypic target adaptations rather than external variables alone. who demonstrated that genetic variations in faba bean lines dictate clear differences in developmental macro‐phenology, flower‐to‐pod conversion dynamics, and baseline anti‐nutritional or bioactive chemical matrices. In another study, 11 fava bean genotypes were used and the amount of L‐DOPA in the flower parts was determined. As a result of the study, the amount of L‐DOPA in the flowers varied between 6.2–9.17 mg/100 g (Bozoğlu and Bezmen 2021). Another study revealed a high degree of variation in L‐DOPA concentration in leaves and flower tissues among six faba bean varieties. In this study, the average L‐DOPA concentration in flowers ranged from 27.8 to 63.5 mg/g based on dry weight (DW), while in leaves tissues it ranged from 18.2 to 48.7 mg/g. No significant correlation was found between L‐DOPA concentrations in flower and leaves tissues (Hu et al. 2015).

The biochemical profiling of *Vicia faba* L. reveals a sophisticated reservoir of antioxidant compounds, including phenolics, carotenoids, anthocyanins, and tocopherols (Altemimi et al. 2017). As the pharmaceutical industry increasingly turns to the botanical kingdom for structural diversity and expansive pharmacological profiles (Ingle et al. 2017), understanding the modulation of phytochemicals like flavonoids, alkaloids, and terpenes (Misra et al. 2024) becomes essential for human nutrition and the mitigation of chronic pathologies (Tiwari et al. 2015). Our findings demonstrate that the accumulation of these metabolites is a highly regulated adaptive response to environmental perturbations. Secondary metabolites, particularly phenolics and salicylic acid, are pivotal regulatory elements in plant ontogeny and stress orchestration (Kumar et al. 2023).

In our study, the significant modulation of TPC in foliar tissues by salt and selenium (*p* < 0.01) aligns with the role of these aromatic metabolites in facilitating physiological resilience against suboptimal conditions. We observed that while mild salinity (Salt 1) triggered peak TPC in leaves and fruits, severe stress (Salt 3) maximized concentrations in the inflorescence. This shift mirrors the specialized adaptive response where plants stabilize essential physiological processes through polyphenols, flavonoids, and phenolic acids to counteract ROS‐induced oxidative damage (Pratyusha 2022). The multifaceted biochemical functionalities of these compounds—serving as antioxidants, antimutagenic, and antimicrobial agents (Kartal et al. 2020) are particularly vital in legumes, which are indispensable for mitigating oxidative damage in biological systems (Lee et al. 2021). Our observation of declining TFC levels toward physiological maturity (from flower to pod) is strongly corroborated by germplasm characterizations in Tunisia (Chaieb et al. 2011). This phenological regulation suggests that flavonoids, as integral components of the plant's defense system (Do et al. 2014), are predominantly sequestered in younger, more vulnerable tissues to neutralize reactive free radicals (Samatha et al. 2012). Exogenous applications of salicylic acid (SA) and selenium (Se) function as potent elicitors of stress resilience in *Vicia faba* L. (Anaya et al. 2017; Soliman and Abdelhameed 2025). Our data supports the hypothesis that Se supplementation facilitates metabolic homeostasis under saline conditions. Specifically, low‐dose Se application restored physiological parameters, a phenomenon also observed where Se enhanced shoot length, fresh biomass, and photosynthetic rate under heavy metal toxicity (Soliman and Abdelhameed 2025). However, the efficacy of Se is biphasic; while concentrations up to 5.0 mg L^−1^ augment productivity (El‐Sherpiny and Kany 2023), supra‐optimal levels can exacerbate oxidative stress and compromise root homeostasis (Mroczek‐Zdyrska and Wójcik 2012). This dose‐dependent response, where low doses (10 mg/L and below) help plants maintain functional homeostasis by modulating stress‐signaling genes like RuBisCO and SOS1 (Farag et al. 2022), is consistent with our findings where Se1 and Se2 doses optimized metabolite profiles.

*Vicia faba* is unique among legumes for containing significant amounts of L‐DOPA (Topal and Bozoğlu 2016; Tesoro et al. 2022), a primary precursor for restoring neurotransmitter homeostasis in PD (Min et al. 2015; Muñoz et al. 2025). Our results showed that L‐DOPA content in all organs was significantly affected by salt and selenium (*p* < 0.05). Under severe salinity (Salt III), L‐DOPA concentrations peaked in floral tissues, suggesting a role as an organic osmolyte or potent antioxidant safeguard (Etemadi et al. 2018). The biosynthesis of L‐DOPA through the shikimic acid pathway is intrinsically linked to the plant's defense architecture (Soares et al. 2014; Shetty et al. 2005). Our data, showing high genotypic variability in pod L‐DOPA levels (particularly in the ‘Lazer’ variety), aligns with studies identifying a broad spectrum of L‐DOPA concentrations across different fava bean accessions (Topal and Bozoğlu 2016; Ryu et al. 2017; Hu et al. 2015). Molecular docking results revealed that levodopa forms stable and efficient interactions with NAD(P)H oxidase (−6.7 kcal/mol). This interaction, stabilized by hydrogen bonds with residues like ASP282 and THR9, supports the potential of L‐DOPA to modulate ROS production at the molecular level. This molecular stability underpins the high antioxidant activity observed in our study, where flowers exhibited the highest DPPH inhibition (78.9%), followed by leaves and fruits. These results confirm previous reports that fava bean flowers possess higher phenolic and antioxidant content than other organs (Akbel et al. 2022). Furthermore, the superior radical scavenging activity of young leaves compared to mature tissues (Duan et al. 2021) and the bioactivity found in pods (Elbadrawy and Mostafa 2024) highlight the potential of using these botanical matrices as substrates for contemporary medicinal compounds (Ingle et al. 2017) and functional foods (Rybiński et al. 2019; Dhull et al. 2022; Elbadrawy and Mostafa 2024).

The accumulation of secondary metabolites in *Vicia faba* L. represents a sophisticated and dynamic adaptive strategy in response to environmental perturbations. Our findings demonstrate that Selenium (Se) functions as a potent metabolic elicitor rather than a mere micronutrient, significantly modulating the biosynthesis of phenolic compounds and L‐DOPA. At optimized low dosages, specifically Se1 and Se2, selenium facilitates the upregulation of the phenylpropanoid biosynthetic pathway, allowing the plant to maintain a robust antioxidant defense system even under saline stress. This is evidenced by the significant modulation of Total Phenolic Content (TPC) and Total Flavonoid Content (TFC) in foliar tissues, which helps the plant mitigate reactive oxygen species (ROS) production and maintain metabolic homeostasis instead of undergoing stress‐induced senescence.

A critical observation in this study is the organ‐specific shift in metabolite allocation, particularly under severe salinity conditions (Salt III). While the plant maintains higher antioxidant levels in vegetative tissues under mild stress, it undergoes a strategic “source‐to‐sink” translocation as stress intensifies, prioritizing the accumulation of L‐DOPA and phenolic antioxidants in the flowers. This prioritized biosynthesis in reproductive organs suggests a “reproductive safeguarding” mechanism intended to protect the plant's genetic progeny from ROS‐induced damage. Furthermore, the observed fluctuations in metabolite levels across different treatments reflect a dose‐dependent, biphasic response to selenium, where low concentrations act as bio‐stimulants while higher doses (S4) under extreme salinity may interfere with primary metabolism or exacerbate oxidative stress. The empirical findings are scientifically validated at the molecular level through docking analysis, which provides a mechanistic framework for the observed antioxidant potency. The thermodynamically favorable binding energy of −6.7 kcal/mol between L‐DOPA and NAD(P)H oxidase indicates that L‐DOPA acts as a natural inhibitor of this pro‐oxidant enzyme. By stabilizing within the redox‐active region of the enzyme through hydrogen bonds and hydrophobic interactions, L‐DOPA effectively limits the initiation of ROS cascades. The strong binding affinity (−6.7 kcal/mol) of L‐DOPA toward NAD(P)H oxidase provides a computational validation for the enhanced in vivo antioxidant capacity observed in our study. NAD(P)H oxidase is a primary enzymatic source of intracellular reactive oxygen species (ROS) under salinity; therefore, its competitive inhibition by L‐DOPA effectively mitigates the ROS cascade, complementing the direct radical scavenging activity measured via DPPH assays (Soares et al. 2014; Tuteja 2007). This molecular stability directly supports the high radical scavenging activity (%DPPH) recorded in the study, confirming that tissues with the highest L‐DOPA content, such as the flowers, possess superior antioxidant capacity to counter salinity‐induced oxidative disequilibrium. While primary growth parameters are common indicators of salinity tolerance, the present study prioritizes the metabolic plasticity of *Vicia faba* . The synthesis of secondary metabolites often precedes visible morphological changes under stress. Specifically, the focus on L‐DOPA—a specialized bioactive compound—provides a more nuanced understanding of how faba beans reprogram their secondary metabolism to maintain cellular integrity. Although specific antioxidant enzymes were not directly measured, the integration of molecular docking with NAD(P)H oxidase provides a viable alternative to explain the biochemical suppression of ROS generation, bridging the gap between total phenolic accumulation and specific enzymatic inhibition.

In this study, we transitioned from general screenings to highly specific analyses by utilizing HPLC‐UV to quantify L‐DOPA, a specialized non‐protein amino acid that serves as a critical defense marker in *Vicia faba* . Although specific antioxidant enzymes were not directly assayed, the integration of molecular docking analysis provides a mechanistically meaningful framework for explaining the suppression of oxidative stress. The thermodynamically favorable binding of L‐DOPA to NAD(P)H oxidase (—6.7 kcal/mol) —a key enzymatic source of intracellular ROS—suggests a direct inhibitory role that limits radical formation at the molecular level. This specific interaction validates that the observed increases in total phenolics and flavonoids are synchronized with a targeted biochemical defense aimed at maintaining redox homeostasis under salinity.

The multivariate clustering pattern observed in PCA provides an integrative perspective that complements the univariate ANOVA findings. The salinity‐driven separation along PC1 indicates that, despite the absence of significant main effects in some univariate comparisons, salinity acts as the principal coordinator of the overall biochemical phenotype. Transitioning the focus from isolated parameters to integrated multivariate clustering profiles aligns with the methodological frameworks established by Granato et al. (2018), who demonstrated that the combined implementation of PCA and HCA offers an indispensable holistic viewpoint to accurately map bioactive compound dynamics and functional properties in dietary matrices. In this context, the orthogonal arrangement of pod‐related vectors (TPC, TFC, AOA, L‐DOPA in pods) relative to leaf and flower vectors strongly supports the hypothesis that pod biochemistry is regulated through pathways partially decoupled from those operating in vegetative and reproductive tissues, pointing to a strictly tissue‐specific regulatory architecture of the shikimate–phenylpropanoid pathway in *Vicia faba* L. Furthermore, the structural clustering of severe‐stress samples with floral metabolite vectors explicitly aligns with the ‘reproductive prioritization hypothesis’ proposed for legumes under abiotic stress (Etemadi et al. 2018), where the plant invests defense resources preferentially in seed‐bearing organs to ensure reproductive success. This clear demarcation of the severe stress‐induced “reproductive‐shifted” profiles finds robust procedural backing in established legume chemometrics, as Abdi and Williams (2010) highlighted that hierarchical agglomerative clustering utilizing Ward's minimum‐variance linkage on Euclidean distances is uniquely powerful at uncovering latent environmental grouping behaviors in complex biological datasets without introductory sorting criteria.

### 4.1 Highlights

– Selenium (Se) application effectively mitigates salinity‐induced oxidative stress in faba bean (*Vicia faba* L.).
– Salt stress at 8 dS/m combined with 0.15 mg/L Se maximizes
– L‐DOPA accumulation in specific plant tissues.
– Plants prioritize reproductive organs (flowers) for antioxidant and L‐DOPA sequestration to ensure reproductive success under high salinity.
– Molecular docking reveals that L‐DOPA acts as a stable inhibitor of NAD(P)H oxidase, with a favorable binding energy of −6.7 kcal/mol.
– Optimized Se dosages (Se1 and Se2) act as biochemical elicitors to enhance the nutraceutical density of the ‘Lazer’ variety.

### 4.2 Study Limitations and Future Perspectives

Despite providing significant insights into the metabolic reprogramming of *Vicia faba* under stress, this study has limitations that open clear avenues for future inquiry. Our analysis focused primarily on biochemical and molecular parameters; however, the absence of longitudinal biomass data prevents a full assessment of how these metabolic shifts translate into overall productivity. Additionally, while molecular docking provided a robust mechanistic framework for explaining ROS suppression, direct assays of antioxidant enzymes such as SOD and CAT were not performed.

Furthermore, the experiments were conducted under controlled greenhouse conditions. While this minimized environmental noise, the results may not be directly generalizable to open‐field environments where fluctuating climate variables influence Selenium (Se) uptake and salinity dynamics. To build upon these findings, future research should prioritize the following areas:

#### 4.2.1 Genomic and Transcriptomic Mapping

A deeper exploration of SULTR transporter genes and SOS1/NHX1 expression pathways is needed to fully map the molecular crosstalk between Se and salt tolerance.

#### 4.2.2 Long‐Term Field Trials

Transitioning to multi‐year studies in naturally saline environments is essential to standardize Se application protocols for commercial use.

#### 4.2.3 In Vivo Bioavailability

Future research should investigate the bioavailability of L‐DOPA and phenolic compounds from Se‐enriched Faba beans in animal models to confirm their therapeutic efficacy against Parkinson's Disease.

#### 4.2.4 By‐Product Upcycling

Investigating the industrial extraction of bioactive metabolites from pods—currently considered agricultural waste—could support the development of high‐value functional food additives.

#### 4.2.5 Synergistic Elicitors

Exploring the combined effects of Se with other phytohormones, such as Salicylic Acid, may provide even more robust protection against extreme abiotic perturbations.

## 5 Conclusions

This research substantiates the role of *Vicia faba* L. as a critical reservoir of bioactive compounds with significant pharmacological potential. Our findings conclude that:

1. Tissue‐Specific Responses: The plant prioritizes reproductive organs (flowers) for antioxidant accumulation under high salinity, ensuring reproductive success through metabolic investment.
2. Optimized Elicitation: Low‐dose selenium supplementation (Se1/Se2) acts as a strategic biostimulant, effectively upregulating TPC, TFC, and L‐DOPA biosynthesis to mitigate salinity‐induced phytotoxicity.
3. L‐DOPA as a Defense Marker: L‐DOPA functions not only as a therapeutic agent for human health but also as a vital component of the plant's stress‐response framework, particularly in the ‘Lazer’ genotype
4. Nutraceutical Valorization: The high antioxidant potency of flowers and leaves, alongside the bioactivity of agricultural by‐products like pods, offers significant opportunities for the development of high‐value functional foods and nutraceuticals.

In conclusion, the integration of Se applications offers a fundamental agricultural approach to enhance both the environmental resilience and nutrient density of faba bean plants in saline agricultural areas.

## Author Contributions

**Nurdoğan Topal:** conceptualization, methodology, formal analysis, writing – review and editing, writing – original draft, investigation, validation, resources, funding acquisition, visualization, software, project administration, supervision, data curation. **Ayşe Topuz:** investigation, data curation.

## Funding

The authors have nothing to report.

## Conflicts of Interest

The authors declare no conflicts of interest.

## Data Availability

The data that support the findings of this study are available from the corresponding author upon reasonable request.
